# Author Correction: Hydrogel dressings with intrinsic antibiofilm and antioxidative dual functionalities accelerate infected diabetic wound healing

**DOI:** 10.1038/s41467-025-64329-7

**Published:** 2025-10-10

**Authors:** Dicky Pranantyo, Chun Kiat Yeo, Yang Wu, Chen Fan, Xiaofei Xu, Yun Sheng Yip, Marcus Ivan Gerard Vos, Surendra H. Mahadevegowda, Priscilla Lay Keng Lim, Liang Yang, Paula T. Hammond, David Ian Leavesley, Nguan Soon Tan, Mary B. Chan-Park

**Affiliations:** 1 Centre for Antimicrobial Bioengineering, School of Chemistry, Chemical Engineering and Biotechnology, Nanyang Technological University, Singapore, Singapore; 2 Antimicrobial Resistance Interdisciplinary Research Group, Singapore-MIT Alliance for Research and Technology, Singapore, Singapore; 3 NTU Institute for Health Technologies, Interdisciplinary Graduate School, Nanyang Technological University, Singapore, Singapore; 4 Skin Research Institute of Singapore, Agency for Science, Technology and Research (A*STAR), Singapore, Singapore; 5 Wenzhou Institute, University of Chinese Academy of Sciences, Wenzhou, Zhejiang China; 6 Lee Kong Chian School of Medicine, Nanyang Technological University, Singapore, Singapore; 7 School of Medicine, Southern University of Science and Technology, Shenzhen, China; 8 Department of Chemical Engineering, Massachusetts Institute of Technology, Cambridge, MA USA; 9 School of Biological Sciences, Nanyang Technological University, Singapore, Singapore

**Keywords:** Gels and hydrogels, Gels and hydrogels, Biomedical materials

Correction to: *Nature Communications* 10.1038/s41467-024-44968-y, published online 01 February 2024

In the original version of the article, an error in Fig. 4c resulted in a duplicated image being included. This did not affect the conclusions. Separately from this, some discussion of the maturation of keratinocytes used imprecise language, and although this did not affect the main conclusions, claims regarding keratinocyte differentiation and maturation have been moderated. Separately to this, analysis was rerun for sample normalisation, the data for which is presented in Supplementary Table 2. This was due to the presence of outliers and the use of an unsuitable statistical test. Although this did not affect the main conclusions, the data have been replaced and some minor changes have been made in the text. Additionally, some labelling of figures had minor errors, which did not affect the conclusions.

Details:

Figure 4c incorrectly labelled seven immunostaining images, including one image duplication. The correct version of Fig. 4 is:

**Correct Figure:** 
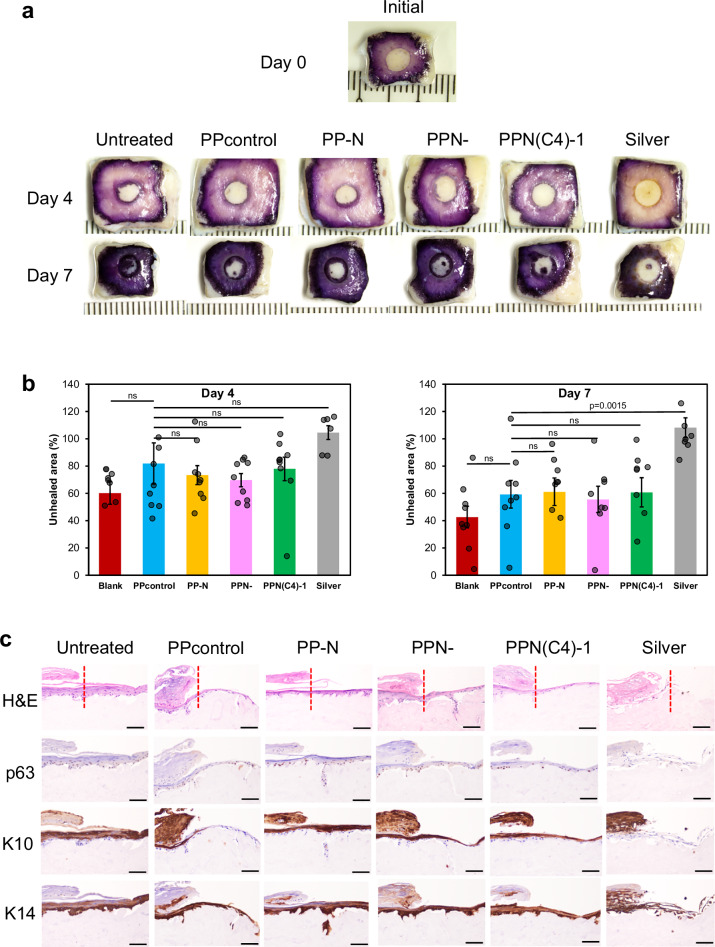


which replaces the incorrect version:

Incorrect Figure:
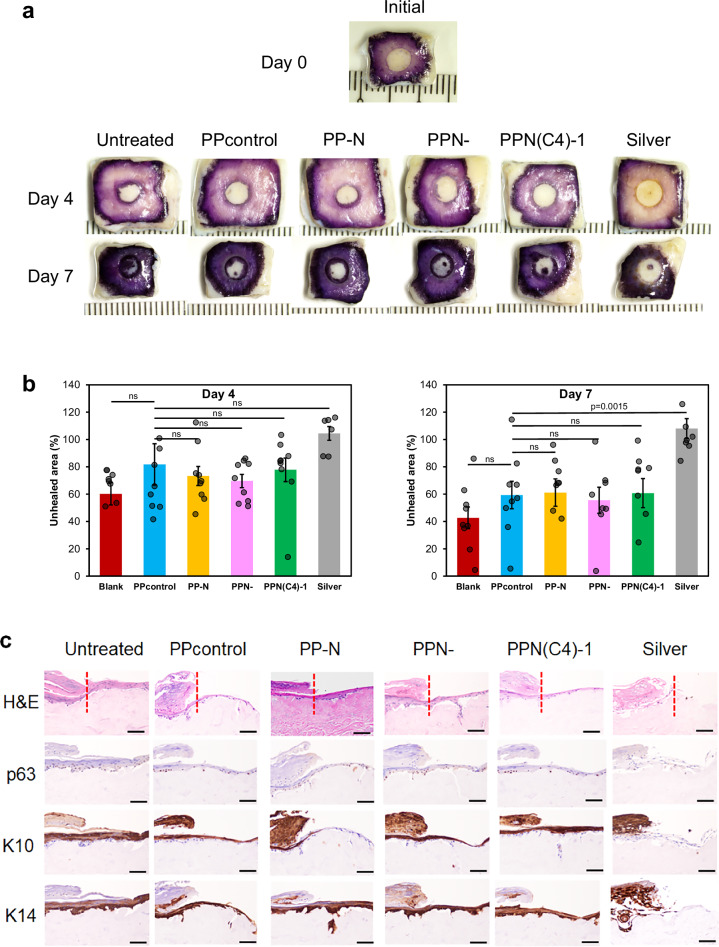


The Abstract incorrectly read ‘Furthermore, a three-dimensional ex vivo human skin equivalent model shows that *N*-acetylcysteine promotes the keratinocyte differentiation and accelerates the reepithelialization process.’ The correct version states ‘Furthermore, a three-dimensional ex vivo human skin equivalent model shows that *N*-acetylcysteine promotes the keratinocyte differentiation.’

The data in Supplementary Table 2 have been reanalysed. The correct version of Supplementary Table 2 is:

**Table:** 

Staining (sample size)	PPcontrol		PP-N			PPN-			PPN(C4)-1		
	Normalized intensity	SEM (±)	Normalized intensity	SEM (±)	*p* value	Normalized intensity	SEM (±)	*p* value	Normalized intensity	SEM (±)	*p* value
H&E (*n* = 10)	1.00	0.09	1.35	0.13	0.04	1.02	0.10	0.89	1.18	0.32	0.60
p63 (*n* = 10)	1.00	0.06	1.18	0.11	0.18	1.20	0.17	0.28	1.21	0.18	0.28
K10 (*n* = 10)	1.00	0.20	2.38	0.42	0.01	1.62	0.35	0.15	2.09	0.44	0.04
K14 (*n* = 10)	1.00	0.13	1.53	0.18	0.03	1.07	0.12	0.72	1.00	0.16	0.98

Which replaces the incorrect version:

**Table:** 

Staining (sample size)	PPcontrol		PP-N			PPN-			PPN(C4)-1		
	Normalized intensity	SEM (±)	Normalized intensity	SEM (±)	*p* value	Normalized intensity	SEM (±)	*p* value	Normalized intensity	SEM (±)	*p* value
H&E (*n* = 10)	1.00	0.07	1.34	0.12	0.03	1.09	0.16	0.64	0.93	0.16	0.67
p63 (*n* = 10)	1.00	0.08	1.17	0.13	0.29	1.25	0.17	0.19	1.29	0.27	0.33
K10 (*n* = 10)	1.00	0.19	4.20	1.91	0.13	1.63	0.38	0.17	2.38	0.54	0.03
K14 (*n* = 10)	1.00	0.17	1.49	0.16	0.05	1.06	0.11	0.76	1.29	0.37	0.49

The results section incorrectly read ‘wounds treated with PP-N exhibited a 34% thicker epidermis than PPcontrol-treated wounds (Supplementary Table 2).’ The correct version reads ‘wounds treated with PP-N exhibited a 35% thicker epidermis than PPcontrol-treated wounds (Supplementary Table 2).’

The Results section incorrectly read ‘We found that DED-HSE treated with PPN- expressed comparable K10 and K14 signal intensities to DED-HSE treated with PPcontrol (Fig. 4c, Supplementary Table 2).’ The correct version reads ‘We found that DED-HSE treated with PPN- expressed comparable K10 and K14 signal intensities (*p* > 0.05) to DED-HSE treated with PPcontrol (Fig. 4c, Supplementary Table 2).’

The Results section incorrectly referenced ‘Fig. 3a,d’ on two occasions, in the 7th and 8th lines of the fourth paragraph. The correct version references ‘Fig. 3a–d’.

The Results section incorrectly read ‘Interestingly, DED-HSE treated with PP-N exhibited a 49% stronger signal intensity for K14 compared to PPcontrol (*p* ≤ 0.05), while showing comparable signal intensity for K10 (Supplementary Table 2),’ The correct version reads ‘Interestingly, DED-HSE treated with PP-N exhibited a 53% stronger signal intensity for K14 compared to PPcontrol (*p* ≤ 0.05), while showing a 138% increase in signal intensity for K10 (*p* ≤ 0.05, Supplementary Table 2),’

The Results section incorrectly read ‘Additionally, DED-HSE treated with PPN(C4)-1 displayed a 138% increase in signal intensity for K10 compared to PPcontrol (*p* ≤ 0.05), with a similar signal intensity observed for K14.’ The correct version reads ‘Additionally, DED-HSE treated with PPN(C4)-1 displayed a 109% increase in signal intensity for K10 compared to PPcontrol (*p* ≤ 0.05), with a comparable (*p* > 0.05) signal intensity observed for K14.’

The Results section incorrectly read ‘To evaluate the potential impact of PP-N, PPN- and PPN(C4)-1 on the maturation of keratinocytes, we also examined the expression of two epithelial-specific differentiation markers.’ The correct version reads ‘To evaluate the potential impact of PP-N, PPN- and PPN(C4)-1 on the maturation of keratinocytes, we also examined the expression of two epithelial-specific markers.’

The Results section incorrectly read ‘The relative distributions of K10 and K14 illustrate the life-cycle of keratinocytes as they transition from proliferating to non-proliferating, differentiated states and repopulate or heal denuded skin.’ The correct version reads ‘The relative distributions of K10 and K14 illustrate the life-cycle of keratinocytes as they transition from proliferating to non-proliferating, differentiated states.’

The Results section incorrectly read ‘This finding indicates that both PP-N and PPN(C4)-1, containing NAC, promote keratinocyte differentiation,’ The correct version reads ‘This finding indicates that PP-N (containing NAC) promotes keratinocyte proliferation,’

The results section incorrectly read ‘and that NAC might enhance the squamous differentiation of keratinocytes.’ The correct version reads ‘and that NAC in the composite PPN(C4)-1 might enhance the squamous differentiation of keratinocytes.’

The caption of Figure 4 incorrectly read ‘b Quantitative measurement of the MTT assay at the day 4 and day 7 time points (triplicated; *n* = 9 biologically independent samples, two-tailed Student’s *t* test, data are presented as mean values ± SEM). c Representative immunostaining images of H&E, p63, Keratin 10 (K10), and Keratin 14 (K14) after treatment with the PPN(C4)−1 hydrogel and its variants (PP-N is without PIM(C4)−1, and PPN- is without NAC). Scale bar = 100 μm.’ The correct version reads ‘b Quantitative measurement of the MTT assay at the day 4 and day 7 time points (triplicated; *n* = 6-9 biologically independent samples, two-tailed Student’s *t* test, data are presented as mean values ± SEM). c Representative immunostaining images of H&E, p63, Keratin 10 (K10), and Keratin 14 (K14) after treatment with the PPN(C4)−1 hydrogel and its variants (PP-N is without PIM(C4)−1, and PPN- is without NAC). The samples were serially sectioned from the same embedded sample, and each sample slice with a section thickness = 4 μm was used only for one type of staining. Scale bar = 100 μm.’

The Results section incorrectly read ‘Wounds treated with PP-N also exhibited reduced expression of VEGF-A and EGF growth factors, although not PDGF-BB and FGF-2,’. The correct version reads ‘Wounds treated with PP-N also exhibited reduced expression of EGF growth factor, although not VEGF-A, PDGF-BB and FGF-2,’.

The Results section incorrectly read ‘The Alg-Com and control Alg fibres did not exhibit bactericidal properties (Fig. 7a–d).’ The correct version reads ‘The Alg-Com and control Alg fibres did not exhibit substantial bactericidal properties (Fig. 7a–d).’

The captions of Figures 7d and 7h which incorrectly read ‘CR-PA after contact incubation with the surface of Alg-PPN(Cn)-0.1 (blue), Alg-PPN(Cn)−1 (green), Alg-PPN(Cn)-5 (orange), and Alg-PPN(Cn)−10 (red) fibres at 37 °C’ The correct version reads ‘CR-PA after contact incubation with the surface of Alg-PPN(Cn)-0.1 (blue), Alg-PPN(Cn)−1 (green), Alg-PPN(Cn)-5 (purple), and Alg-PPN(Cn)−10 (red) fibres at 37 °C’.

The Discussion section incorrectly read ‘Finally, the incorporation of the antioxidant NAC in the PPN hydrogel promotes re-epithelialization and keratinocyte differentiation,’ The correct version reads ‘Finally, the incorporation of the antioxidant NAC in the PPN hydrogel promotes keratinocyte differentiation,’.

The discussion section incorrectly read ‘Uninfected wounds treated with PP-N (hydrogel without PIM but with NAC) exhibited a 34% increase in epidermal thickness’. The correct version reads ‘Uninfected wounds treated with PP-N (hydrogel without PIM but with NAC) exhibited a 35% increase in epidermal thickness’.

The Discussion section incorrectly read ‘indicating that NAC incorporation enhances the squamous differentiation of keratinocytes.’ The correct version reads ‘indicating that NAC incorporation into the composite PPN hydrogel enhances the squamous differentiation of keratinocytes’.

In Figure 1a (ii) ‘PIM(C8)-Mal’ was referred to as ‘PIM(C4)-Mal’.

In Figure 7 (all panels), ‘Alg-Com’ was referred to as ‘Alg-Ag’.

These points have been corrected in both the PDF and HTML versions of the Article.

## Supplementary information

Corrected Supplementary Information

