# Supplementary material for: Hydrogel dressings with intrinsic antibiofilm and antioxidative dual functionalities accelerate infected diabetic wound healing
Source: Nat Commun. 2024 Feb 1;15:954. doi: 10.1038/s41467-024-44968-y (PMC10830466; doi:10.1038/s41467-024-44968-y)
Supplement: Supplementary file 1 — Supplementary Information [file 41467_2024_44968_MOESM1_ESM.docx]

**Supplementary Information**

Hydrogel dressings with intrinsic antibiofilm and antioxidative dual functionalities accelerate infected diabetic wound healing

Dicky Pranantyo^1,2†^, Chun Kiat Yeo^1,3†^, Yang Wu^1^, Chen Fan^4,5^, Xiaofei Xu^1^, Yun Sheng Yip^6^, Marcus Ivan Gerard Vos^6^, Surendra H. Mahadevegowda^1^, Priscilla Lay Keng Lim^4^,

Liang Yang^7^, Paula T. Hammond^2,8^, David Ian Leavesley^4^, Nguan Soon Tan^6,9^*,

Mary B. Chan-Park^1,9^*

^1^ Centre for Antimicrobial Bioengineering, School of Chemistry, Chemical Engineering and Biotechnology, Nanyang Technological University, 62 Nanyang Drive, Singapore 637459

^2^ Antimicrobial Resistance Interdisciplinary Research Group, Singapore-MIT Alliance for Research and Technology, Singapore 138602

^3^ NTU Institute for Health Technologies, Interdisciplinary Graduate School, Nanyang Technological University, Singapore 637553

^4^ Skin Research Institute of Singapore, Agency for Science, Technology and Research (A*STAR), 11 Mandalay Road, Singapore 308232

^5^ Wenzhou Institute, University of Chinese Academy of Sciences, Wenzhou, Zhejiang, China 325000

^6^ Lee Kong Chian School of Medicine, Nanyang Technological University, 59 Nanyang Drive, Singapore 636921

^7^ School of Medicine, Southern University of Science and Technology, Shenzhen, China 518055

^8^ Department of Chemical Engineering, Massachusetts Institute of Technology, Cambridge, MA 02142, USA

^9^ School of Biological Sciences, Nanyang Technological University, 60 Nanyang Drive, Singapore 637551

*E-mail: [mbechan@ntu.edu.sg](mailto:mbechan@ntu.edu.sg) ; [nstan@ntu.edu.sg](mailto:nstan@ntu.edu.sg)

**Supplementary Table 1.** Minimum inhibitory concentrations (MICs) of PIM(C4)-Mal

| Bacteria | MIC (µg/mL) |
| --- | --- |
| *E. faecium* 19434 | 4 |
| MRSA BAA-40 | 2 |
| MRSA USA300 | 4 |
| *E. cloacae* 13047 | 4 |
| *K. pneumoniae* 13883 | 8 |
| PAO1 | 4 |
| CR-PA | 8 |
| CR-AB | 8 |

**Supplementary Table 2.** Normalized signal intensity from immunostaining images on the *ex vivo* DED-HSE model after 7 days.

| Staining (sample size) |  | PPcontrol | |  | PP-N | | |  | PPN- | | |  | PPN(C4)-1 | | |
| --- | --- | --- | --- | --- | --- | --- | --- | --- | --- | --- | --- | --- | --- | --- | --- |
|  |  | Normalized intensity | SEM (±) |  | Normalized intensity | SEM (±) | *p* value |  | Normalized intensity | SEM (±) | *p* value |  | Normalized intensity | SEM (±) | *p* value |
| H&E (n = 10) |  | 1.00 | 0.09 |  | 1.35 | 0.13 | 0.04 |  | 1.02 | 0.10 | 0.89 |  | 1.18 | 0.32 | 0.60 |
| p63 (n = 10) |  | 1.00 | 0.06 |  | 1.18 | 0.11 | 0.18 |  | 1.20 | 0.17 | 0.28 |  | 1.21 | 0.18 | 0.28 |
| K10 (n = 10) |  | 1.00 | 0.20 |  | 2.38 | 0.42 | 0.01 |  | 1.62 | 0.35 | 0.15 |  | 2.09 | 0.44 | 0.04 |
| K14 (n = 10) |  | 1.00 | 0.13 |  | 1.53 | 0.18 | 0.03 |  | 1.07 | 0.12 | 0.72 |  | 1.00 | 0.16 | 0.98 |

Notes: SEM (±) indicates standard error of the mean;

*p* value was obtained by two-tailed Student’s *t*-test in comparison to the PPcontrol treatment group;

Signal intensities are normalized to the PPcontrol treatment group;

For H&E staining, the intensity refers to the epidermal thickness;

For p63, the intensity refers to the number of highly proliferative p63-expressing cells (brown dots) over proliferative and non-proliferative cells (brown plus blue dots);

For K10, the intensity refers to the relative density of K10 (brown) staining, measured using ImageJ;

For K14, the intensity refers to the relative density of K14 (brown) staining, measured using ImageJ.

**Supplementary Table 3.** Leaching rate of PIM(C4) from Alg-PPN(C4) fibers (data obtained from Figures S15a,c and S16a).

| Sample | Initial PIM  (mg) | Leached PIM  (*µ*g) | PIM leach rate per day (%) |
| --- | --- | --- | --- |
| Alg-PPN(C4)-0.1 | 0.166 | 3.415 | 2.057 % |
| Alg-PPN(C4)-1 | 1.635 | 3.573 | 0.219 % |
| Alg-PPN(C4)-5 | 7.673 | 0.733 | 0.010 % |
| Alg-PPN(C4)-10 | 14.252 | 2.844 | 0.020 % |

**Supplementary Table 4.** Leaching rate of NAC from Alg-PPN(C4) fibers (data obtained from Figures S15b,d and S16b).

| Sample | Initial NAC  (mg) | Leached NAC  (*µ*g) | NAC leach rate per day (%) |
| --- | --- | --- | --- |
| Alg-PPN(C4)-0.1 | 0.271 | 1.646 | 0.608 % |
| Alg-PPN(C4)-1 | 0.267 | 2.376 | 0.890 % |
| Alg-PPN(C4)-5 | 0.250 | 1.955 | 0.780 % |
| Alg-PPN(C4)-10 | 0.233 | 2.606 | 1.120 % |

**Supplementary Table 5.** Leaching rate of PEG-4Mal from Alg-PPN(C4) fibers (data obtained from Figures S15e and S16c).

| Sample | Initial PEG  (mg) | Leached PEG  (*µ*g) | PEG leach rate per day (%) |
| --- | --- | --- | --- |
| Alg-PPN(C4)-0.1 | 16.594 | 4.269 | 0.026 % |
| Alg-PPN(C4)-1 | 16.350 | 42.541 | 0.260 % |
| Alg-PPN(C4)-5 | 15.346 | 100.316 | 0.654 % |
| Alg-PPN(C4)-10 | 14.252 | 162.508 | 1.140 % |

**Supplementary Table 6.** Antibacterial activity of the Alg-PPN(C4) fibers based on contact treatment.

| **Sample** | **Log reduction of bacteria** | | | |
| --- | --- | --- | --- | --- |
|  | MRSA | CR-AB | PAO1 | CR-PA |
| Alg | 1.09 | 1.28 | 0.22 | 0.04 |
| Alg-Com | 3.26 | 1.56 | 2.24 | 0.11 |
| Alg-PPN(C4)-0.1 | 2.23 | 1.90 | 2.08 | 1.05 |
| Alg-PPN(C4)-1 | 5.89 | 5.69 | 4.21 | 4.71 |
| Alg-PPN(C4)-5 | 7.56* | 7.12* | 7.72* | 7.48* |
| Alg-PPN(C4)-10 | 7.56* | 7.12* | 7.72* | 7.48* |
| Log of initial inoculum | 7.56 | 7.12 | 7.72 | 7.48 |

**Supplementary Table 7.** Antibacterial activity of the Alg-PPN(C8) fibers based on contact treatment.

| **Sample** | **Log reduction of bacteria** | | | |
| --- | --- | --- | --- | --- |
|  | MRSA | CR-AB | PAO1 | CR-PA |
| Alg | 1.09 | 1.28 | 0.22 | 0.04 |
| Alg-Com | 3.26 | 1.56 | 2.24 | 0.11 |
| Alg-PPN(C8)-0.1 | 2.51 | 3.98 | 2.34 | 3.05 |
| Alg-PPN(C8)-1 | 5.88 | 6.01 | 4.38 | 5.13 |
| Alg-PPN(C8)-5 | 7.56* | 7.12* | 7.72* | 7.48* |
| Alg-PPN(C8)-10 | 7.56* | 7.12* | 7.72* | 7.48* |
| Log of initial inoculum | 7.56 | 7.12 | 7.72 | 7.48 |

**Supplementary Table 8.** Antibacterial activity of the Alg-PPN(C10) fibers based on contact treatment.

| **Sample** | **Log reduction of bacteria** | | | |
| --- | --- | --- | --- | --- |
|  | MRSA | CR-AB | PAO1 | CR-PA |
| Alg | 1.09 | 1.28 | 0.22 | 0.04 |
| Alg-Com | 3.26 | 1.56 | 2.24 | 0.11 |
| Alg-PPN(C10)-0.1 | 2.82 | 4.10 | 2.76 | 3.86 |
| Alg-PPN(C10)-1 | 5.98 | 5.79 | 5.55 | 5.35 |
| Alg-PPN(C10)-5 | 7.56* | 7.12* | 7.72* | 7.48* |
| Alg-PPN(C10)-10 | 7.56* | 7.12* | 7.72* | 7.48* |
| Log of initial inoculum | 7.56 | 7.12 | 7.72 | 7.48 |

* denotes that no bacterial colonies were observed on the agar plate after contact incubation for 1 h.

**Supplementary Table 9.** Biofilm killing effects of the Alg-PPN(C4) fibers based on contact treatment.

| **Sample** | **Log reduction of biofilm colonies** | | | |
| --- | --- | --- | --- | --- |
|  | MRSA | CR-AB | PAO1 | CR-PA |
| Alg | 0.66 | 0.21 | 0.46 | 0.17 |
| Alg-Com | 0.83 | 0.49 | 0.79 | 0.25 |
| Alg-PPN(C4)-0.1 | 1.39 | 0.83 | 0.63 | 0.89 |
| Alg-PPN(C4)-1 | 1.55 | 1.12 | 1.30 | 1.19 |
| Alg-PPN(C4)-5 | 3.01 | 2.69 | 2.16 | 1.77 |
| Alg-PPN(C4)-10 | 3.81 | 3.43 | 3.37 | 3.01 |
| Log of initial biofilm | 8.14 | 8.05 | 8.26 | 8.11 |

**Supplementary Table 10.** Biofilm killing effects of the Alg-PPN(C8) fibers based on contact treatment.

| **Sample** | **Log reduction of biofilm colonies** | | | |
| --- | --- | --- | --- | --- |
|  | MRSA | CR-AB | PAO1 | CR-PA |
| Alg | 0.66 | 0.21 | 0.46 | 0.17 |
| Alg-Com | 0.83 | 0.49 | 0.79 | 0.25 |
| Alg-PPN(C8)-0.1 | 1.84 | 3.90 | 1.62 | 2.26 |
| Alg-PPN(C8)-1 | 3.02 | 4.95 | 3.13 | 4.01 |
| Alg-PPN(C8)-5 | 4.13 | 8.05* | 8.26* | 4.86 |
| Alg-PPN(C8)-10 | 6.80 | 8.05* | 8.26* | 8.11* |
| Log of initial biofilm | 8.14 | 8.05 | 8.26 | 8.11 |

**Supplementary Table 11.** Biofilm killing effects of the Alg-PPN(C10) fibers based on contact treatment.

| **Sample** | **Log reduction of biofilm colonies** | | | |
| --- | --- | --- | --- | --- |
|  | MRSA | CR-AB | PAO1 | CR-PA |
| Alg | 0.66 | 0.21 | 0.46 | 0.17 |
| Alg-Com | 0.83 | 0.49 | 0.79 | 0.25 |
| Alg-PPN(C10)-0.1 | 2.33 | 4.18 | 1.95 | 2.99 |
| Alg-PPN(C10)-1 | 5.17 | 5.79 | 3.59 | 4.82 |
| Alg-PPN(C10)-5 | 8.14* | 8.05* | 8.26* | 8.11* |
| Alg-PPN(C10)-10 | 8.14* | 8.05* | 8.26* | 8.11* |
| Log of initial biofilm | 8.14 | 8.05 | 8.26 | 8.11 |

* denotes that no bacterial colonies were observed on the agar plate after contact incubation for 16 h.

**Supplementary Figure 1.** Preparation of PIM(Cn) and PIM(Cn)-Mal.

**Supplementary Figure 2.** Preparations of (a) PEG-2Mal-2NAC and (b) thiol-grafted alginate (Alg-SH) and PIM-grafted alginate (Alg-SH-PIM).


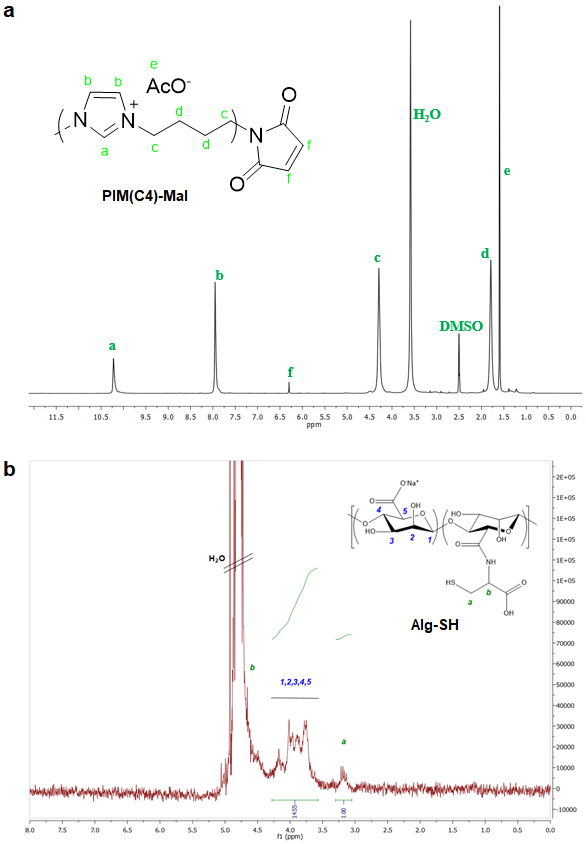


**Supplementary Figure 3.** ^1^H NMR spectra of (a) PIM(C4)-Mal in DMSO-d_6_ and (b) Alg-SH in D_2_O.


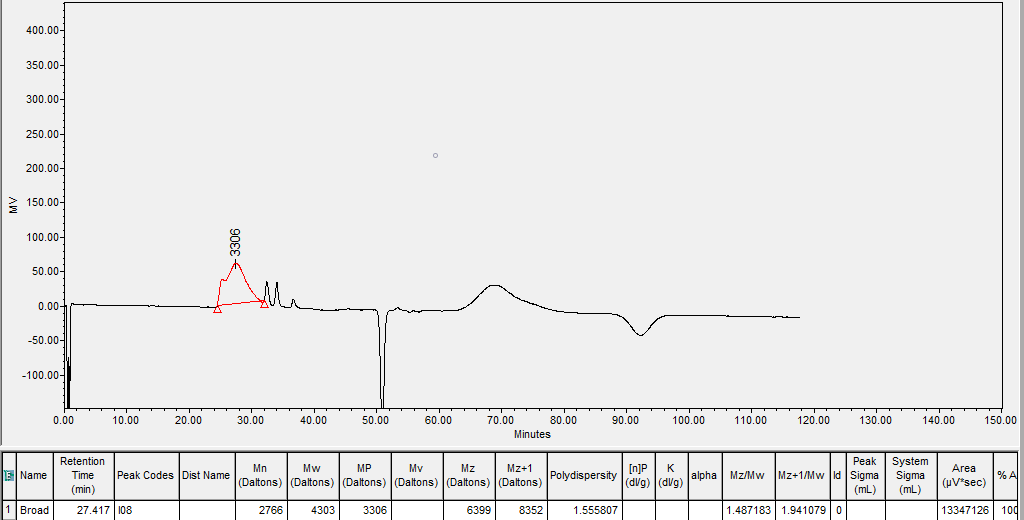


**Supplementary Figure 4.** Molecular weight of PIM(C4)-Mal using gel permeation chromatography (GPC).

**
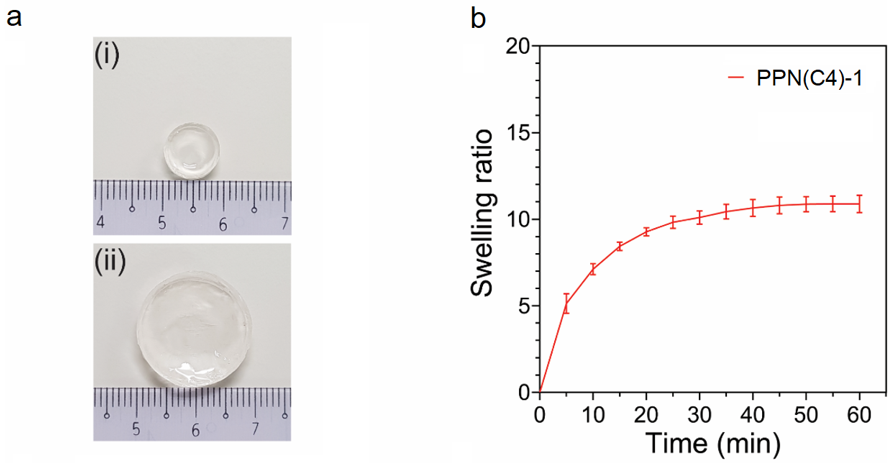
**

**Supplementary Figure 5.** (a) Visual appearance and size of PPN(C4)-1 hydrogels fabricated in (i) 96-well plates and (ii) 24-well plates. (b) Swelling ratio (mass increase/initial mass) versus time of the PPN(C4)-1 hydrogel (n=3 independent experiments, data are presented as mean values ± SD).


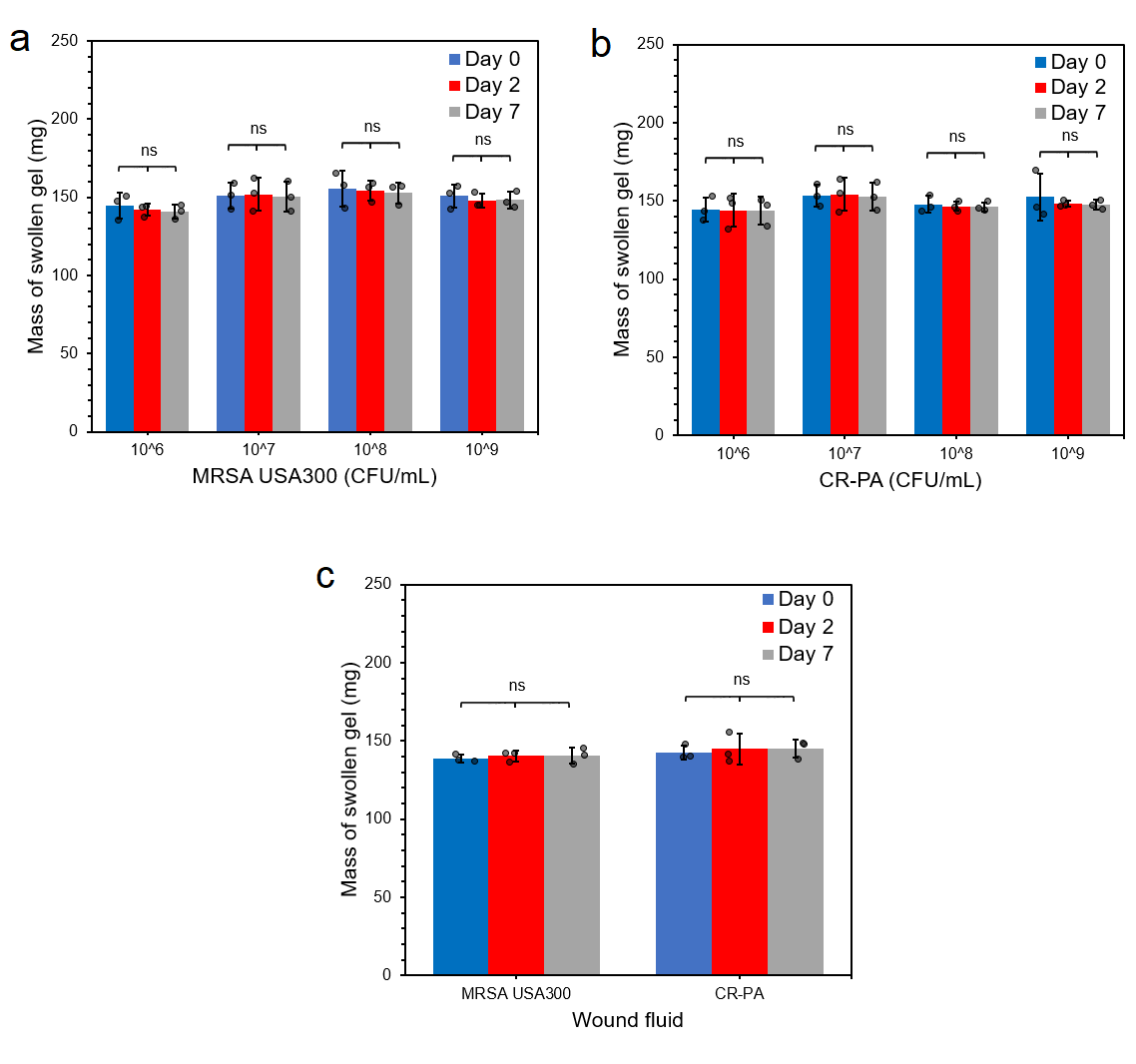


**Supplementary Figure 6.** Mass of swollen PPN(C4)-1 hydrogels when incubated with (a) extract of MRSA USA300, (b) extract of CR-PA, and (c) wound fluids of MRSA USA300- and CR-PA-infected wounds for 2 and 7 days (n=3 independent experiments, two-tailed Student’s *t*-test, data are presented as mean values ± SD).


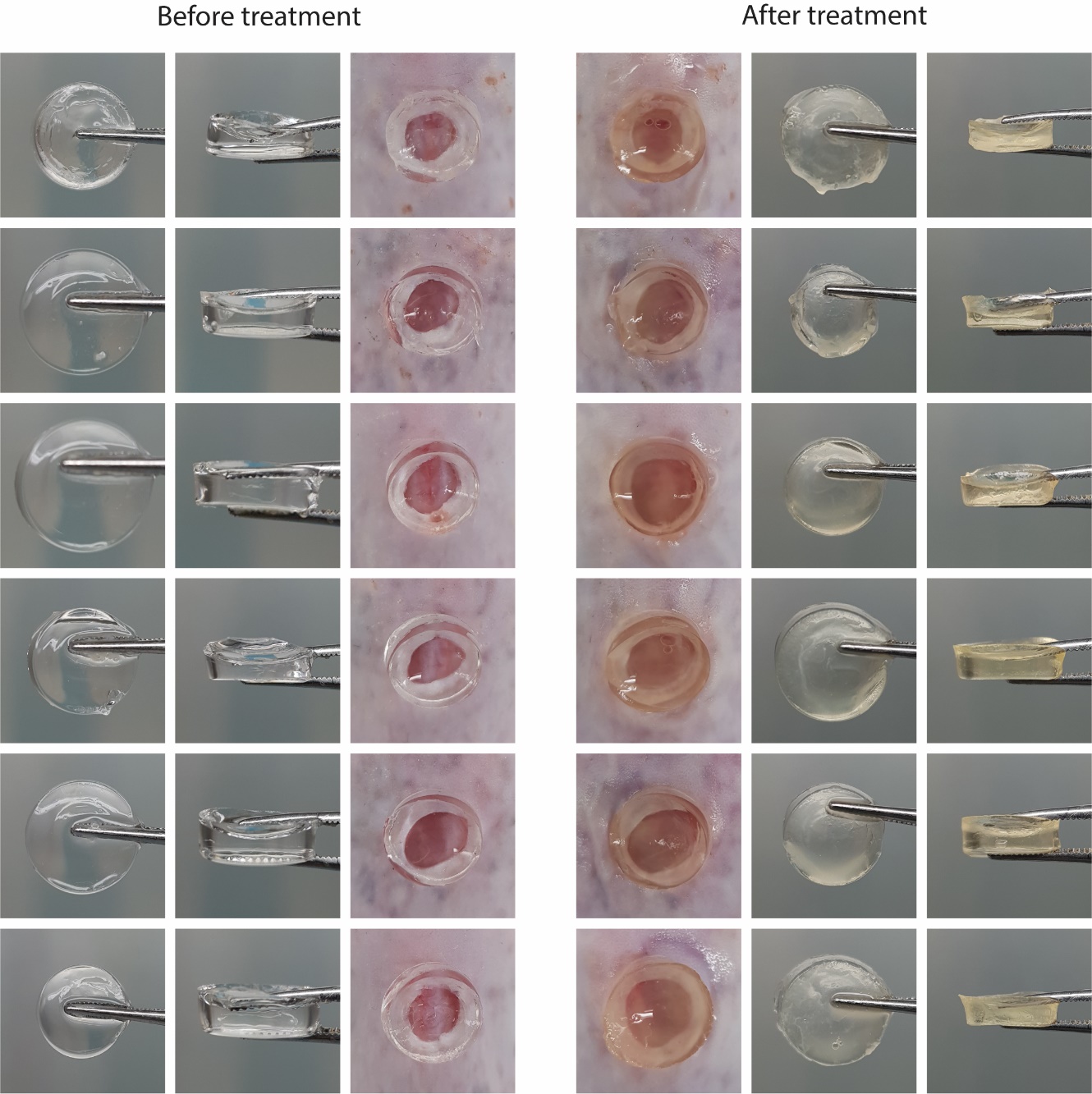


**Supplementary Figure 7.** Visual appearance of PPN(C4)-1 hydrogel before (left) and after (right) 2 days of treatment on MRSA USA300-infected wounds of mice.

**
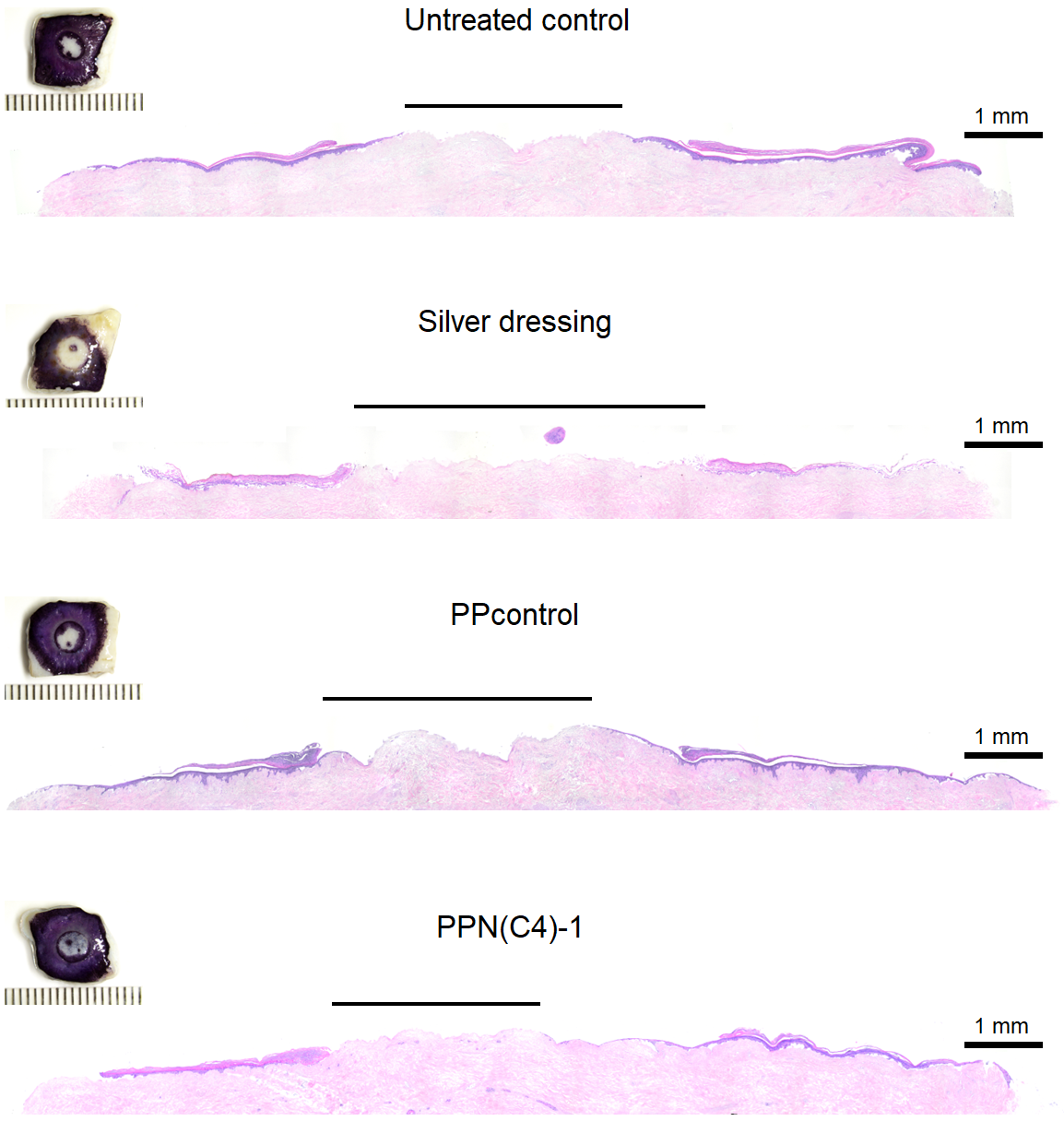
**

**Supplementary Figure 8.** Images of the de-epidermised dermis (DEDs) that were stained with MTT after various treatments for 7 days and their corresponding H&E images. Black lines represent the non-epithelialized part of the DEDs.


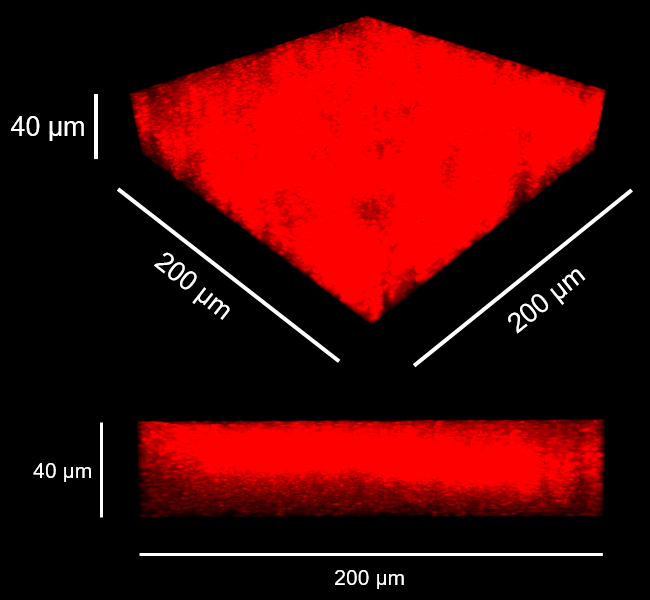

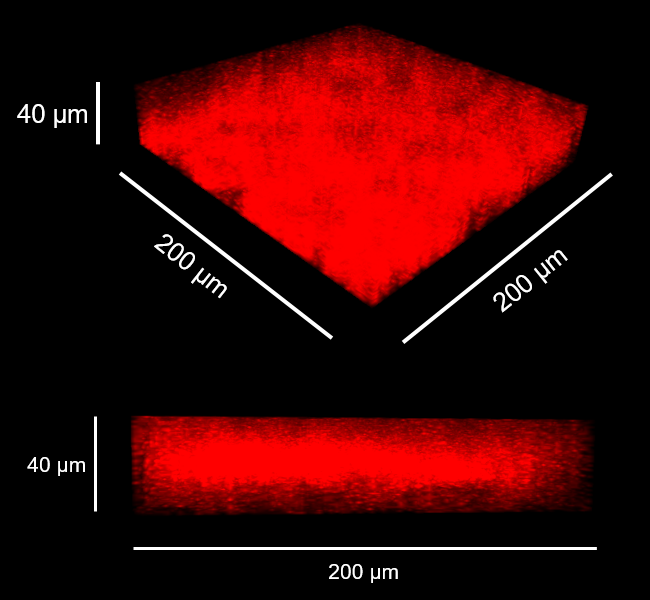

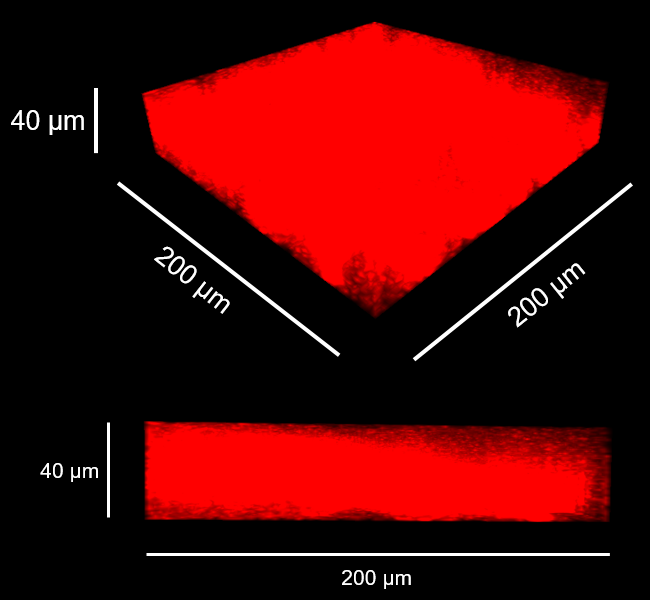

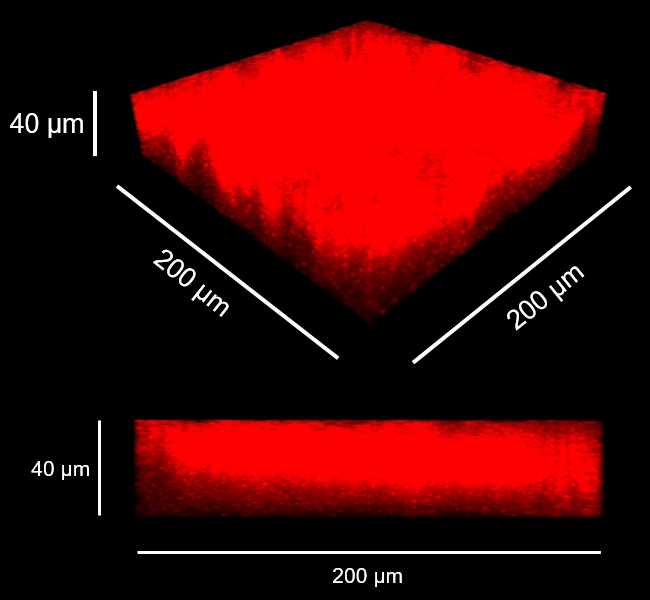


**Supplementary Figure 9a.** Confocal microscopy images showing the 3D and side views of the mCherry-tagged MRSA USA300 biofilm on the wound tissues of the untreated diabetic mice after 2 days post-infection.


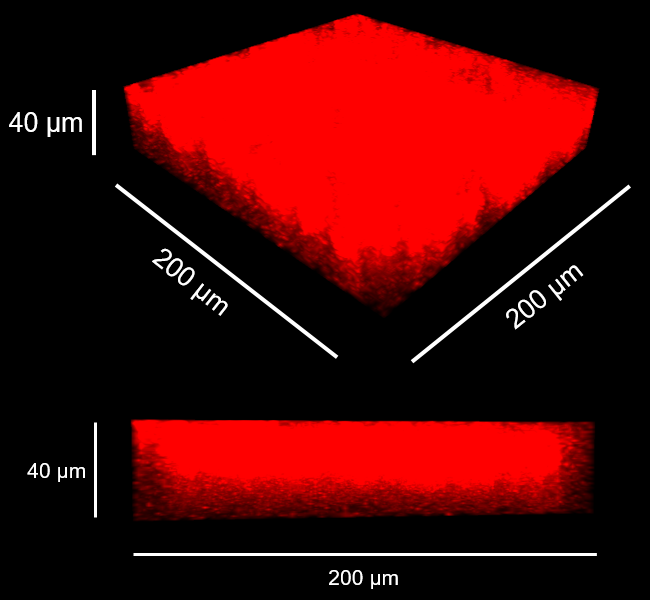

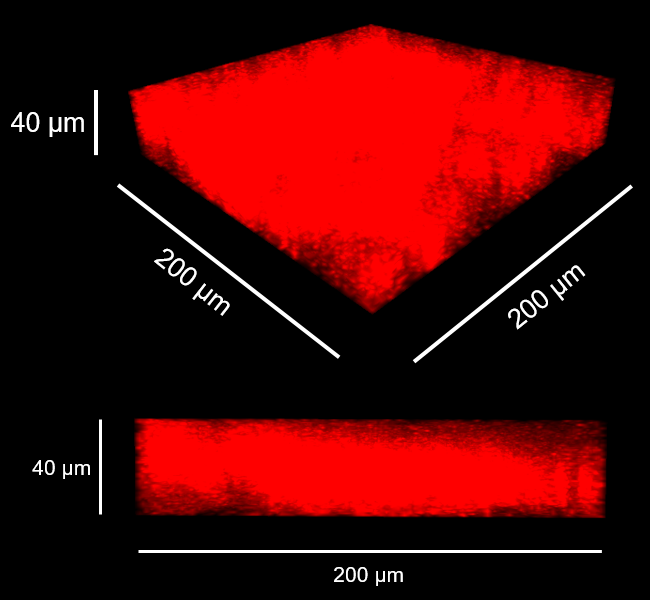

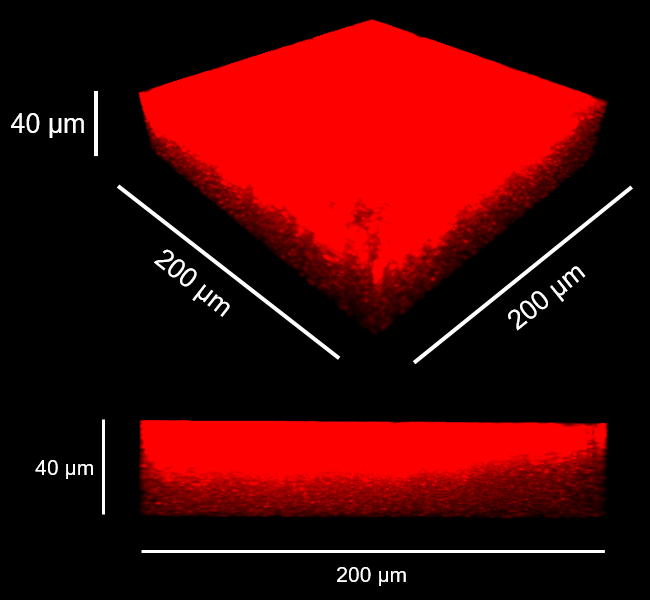

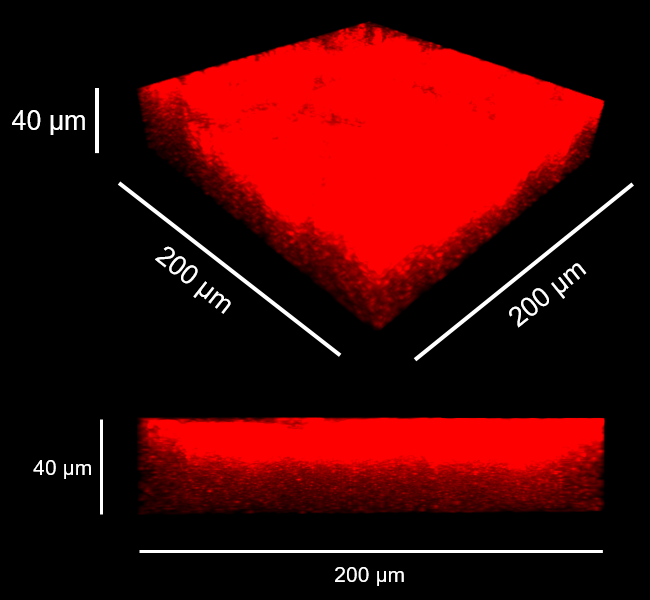


**Supplementary Figure 9b.** Confocal microscopy images showing the 3D and side views of the mCherry-tagged MRSA USA300 biofilm on the wound tissues of the PPcontrol hydrogel-treated diabetic mice after 2 days post-infection.


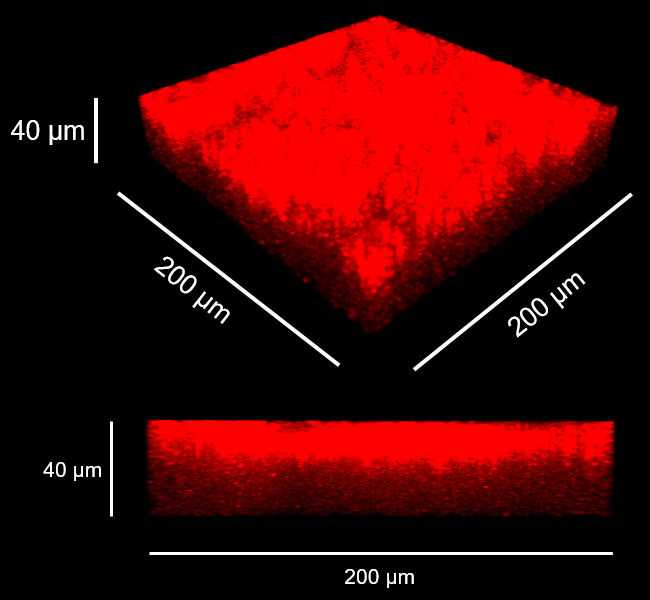

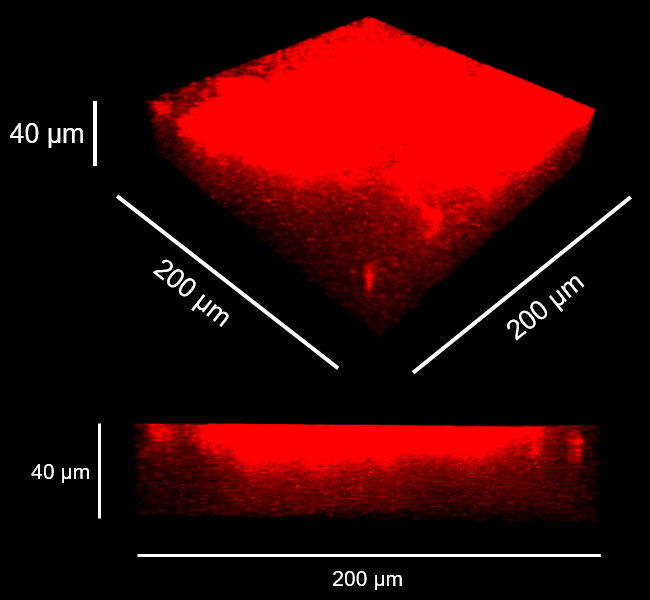

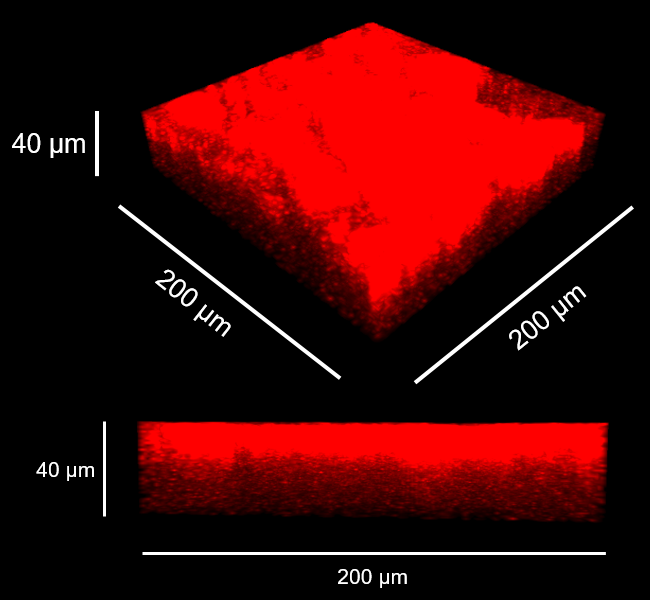

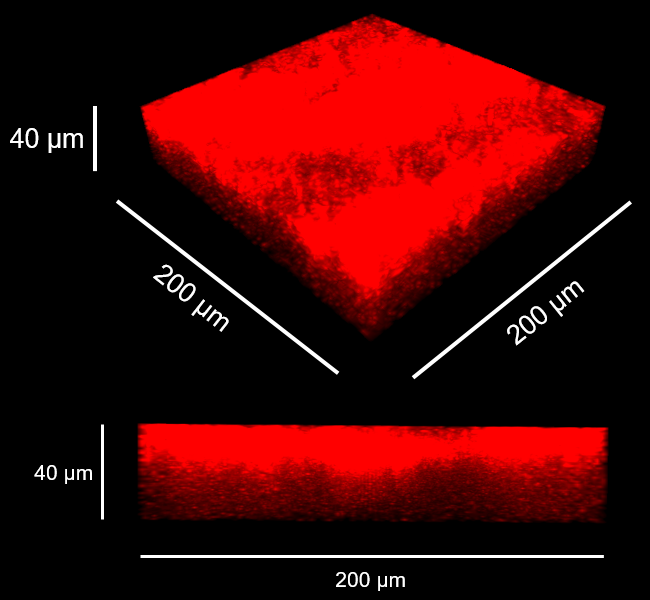


**Supplementary Figure 9c.** Confocal microscopy images showing the 3D and side views of the mCherry-tagged MRSA USA300 biofilm on the wound tissues of the silver hydrogel-treated diabetic mice after 2 days post-infection.


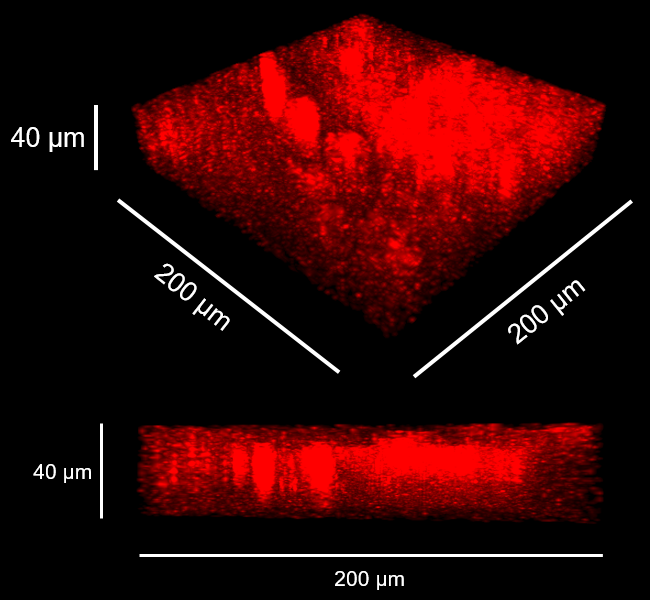

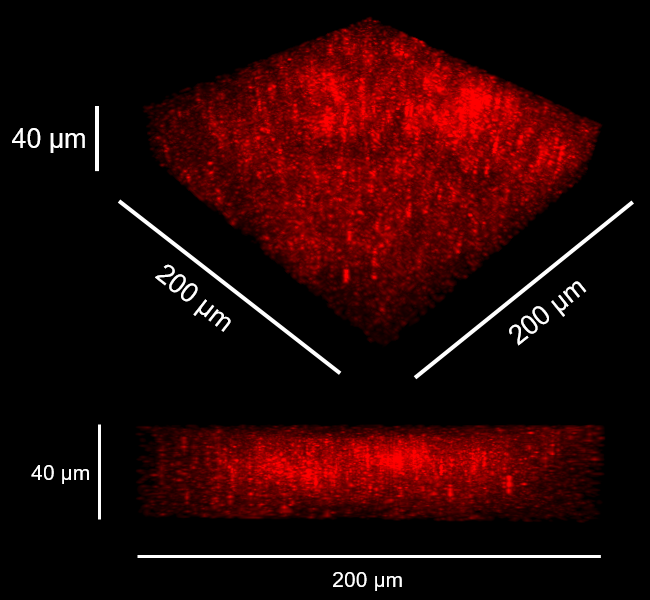

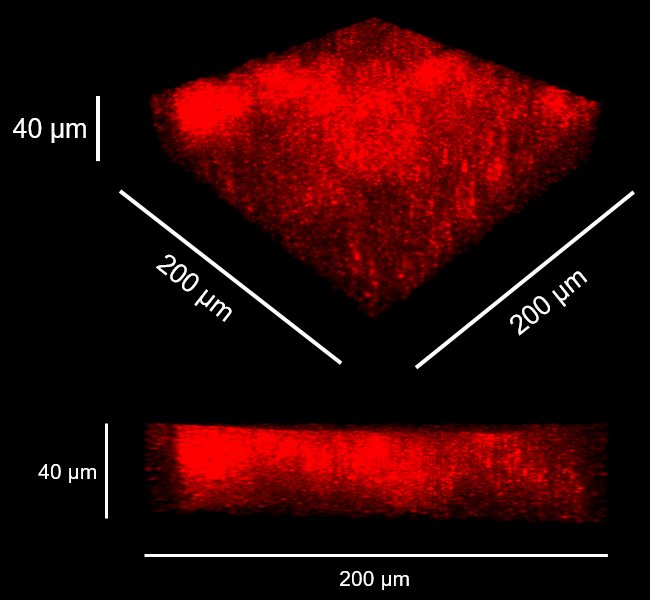

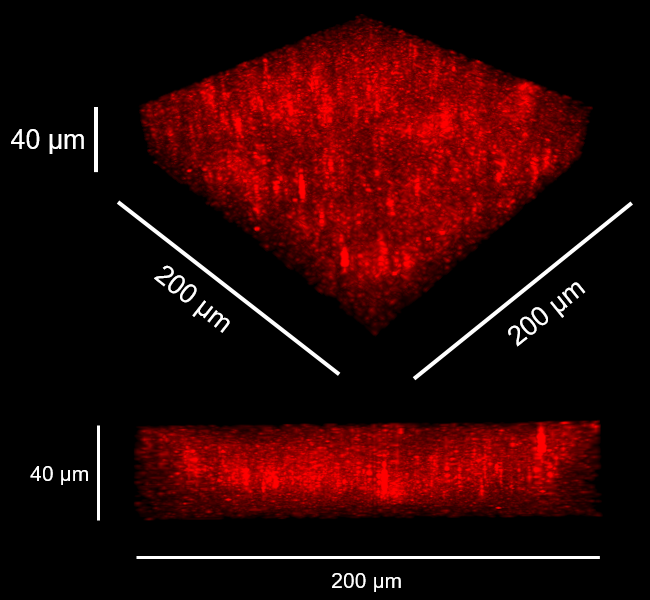


**Supplementary Figure 9d.** Confocal microscopy images showing the 3D and side views of the mCherry-tagged MRSA USA300 biofilm on the wound tissues of the PPN(C4)-1 hydrogel-treated diabetic mice after 2 days post-infection.


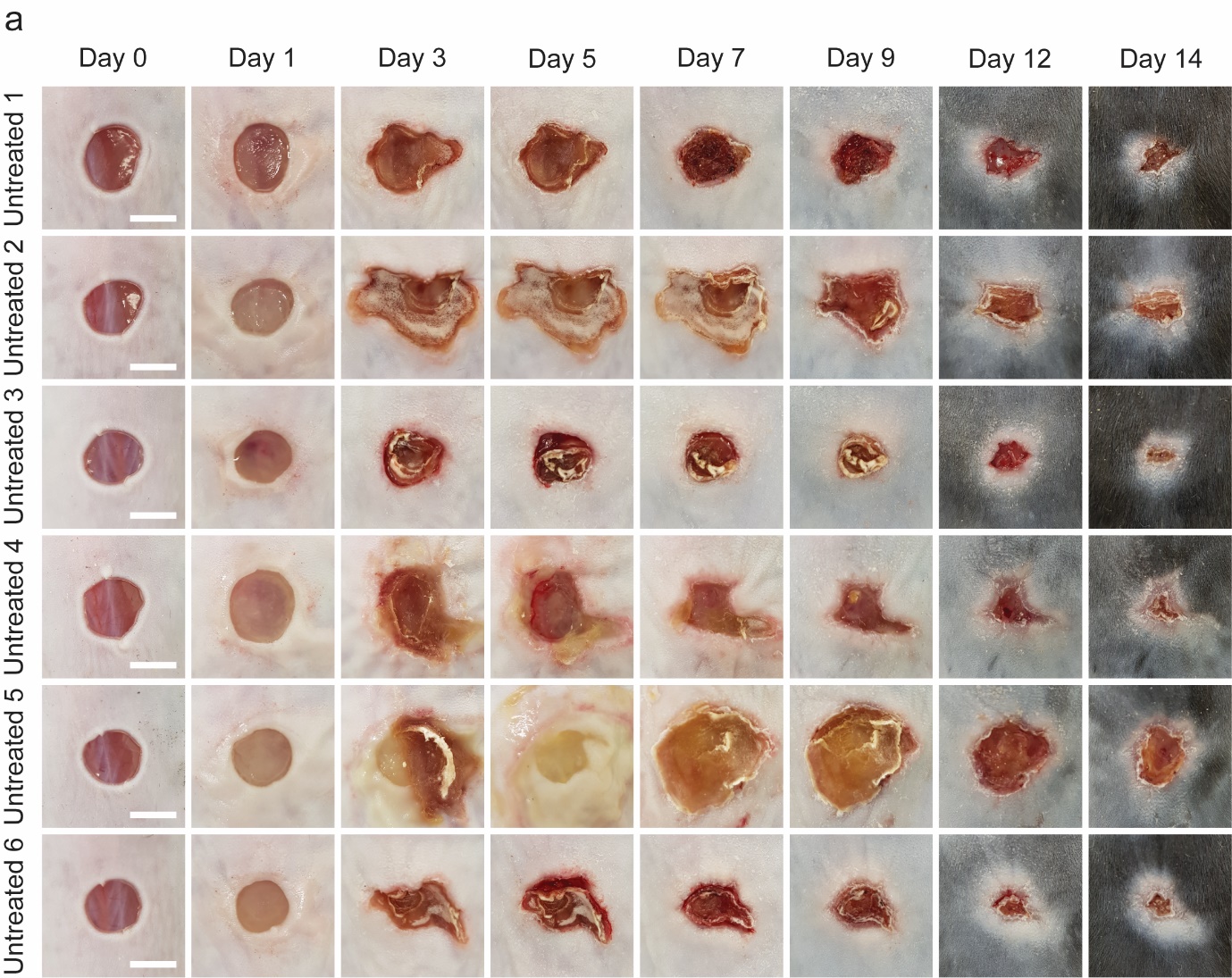


**Supplementary Figure 10a.** Visual appearance of untreated control wounds between dressing changes over 2 weeks on MRSA USA300-infected diabetic mouse wounds. Scale bar = 5 mm. (Six mice were used.)


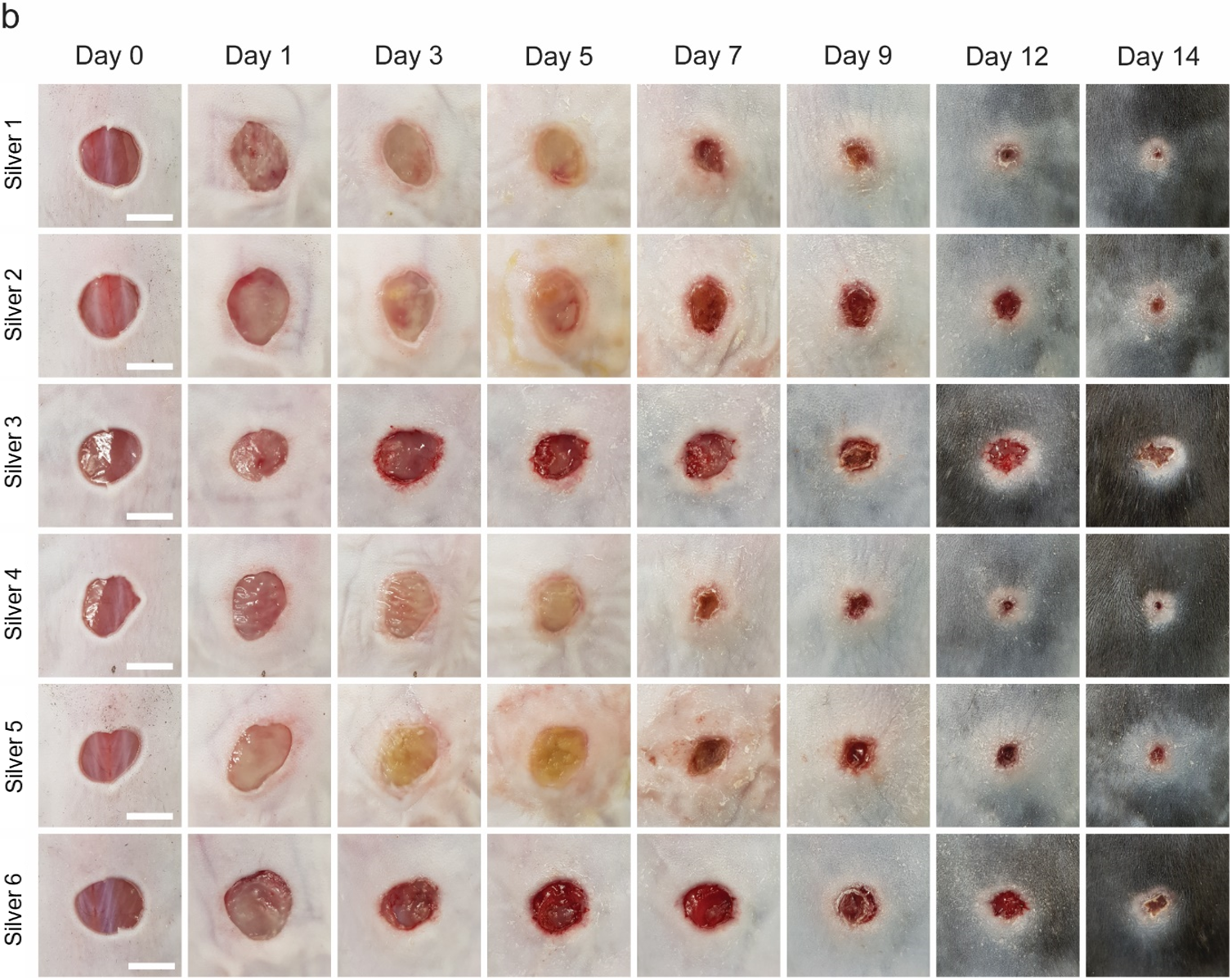


**Supplementary Figure 10b.** Visual appearance of silver dressing-treated wounds between dressing changes over 2 weeks on MRSA USA300-infected diabetic mouse wounds. Scale bar = 5 mm. (Six mice and 1 batch of silver dressing were used.)


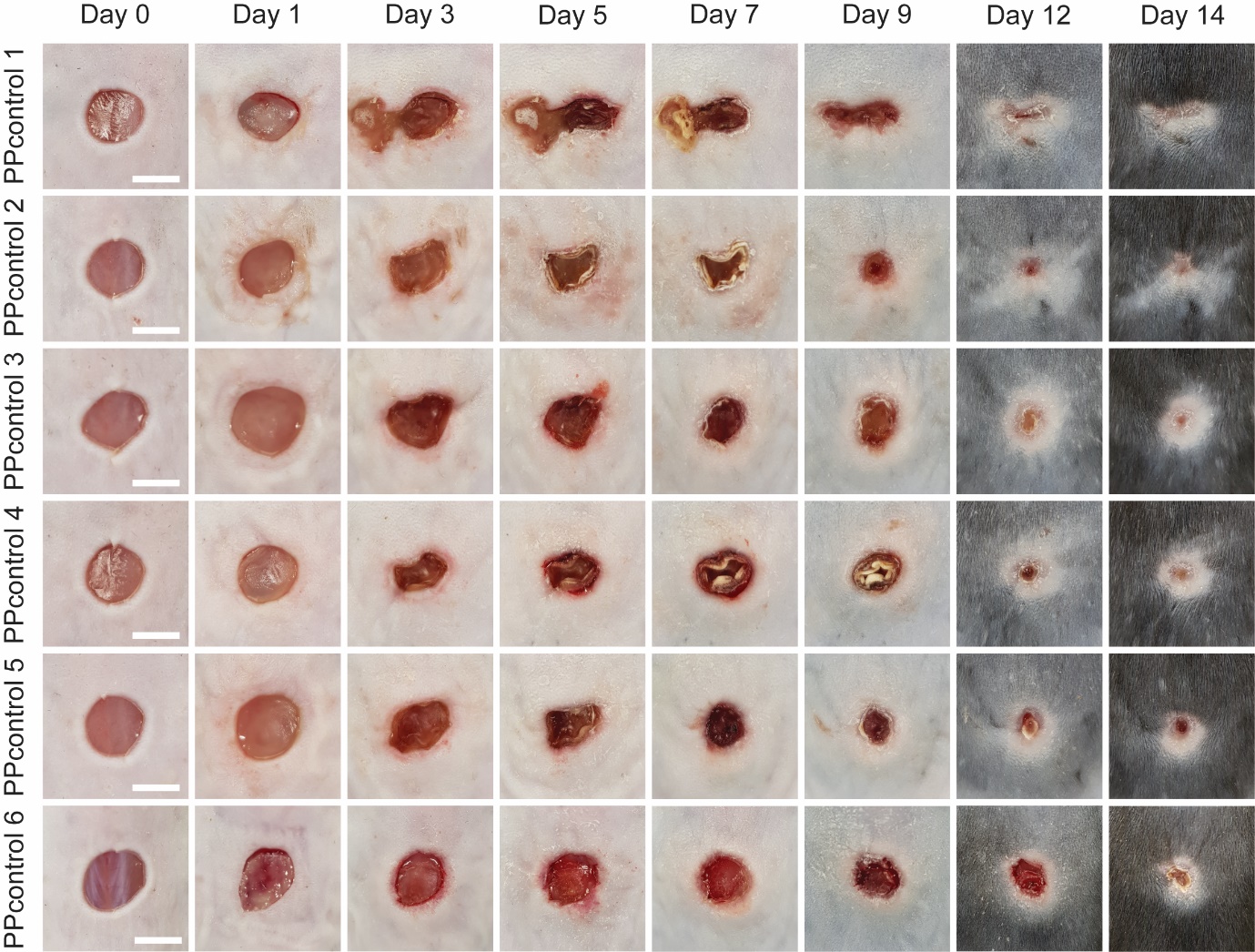


**Supplementary Figure 10c.** Visual appearance of PPcontrol-treated wounds between dressing changes over 2 weeks on MRSA USA300-infected diabetic mouse wounds. Scale bar = 5 mm. (Six mice and 1 batch of polymer were used.)


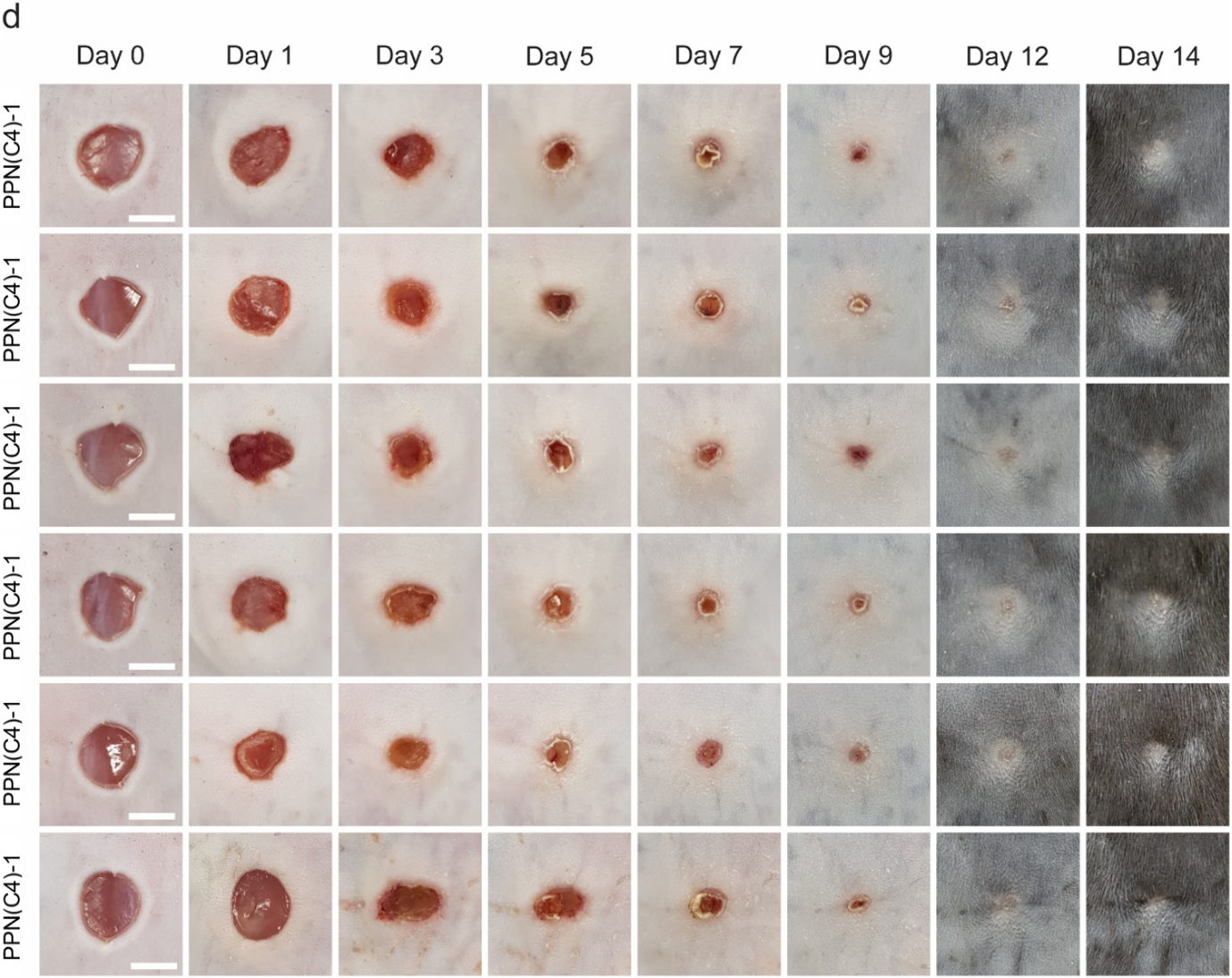


**Supplementary Figure 10d.** Visual appearance of PPN(C4)-1 treated wounds between dressing changes over 2 weeks on MRSA USA300-infected diabetic mice wounds. Scale bar = 5 mm. (Six mice and 1 batch of polymer were used.)

**
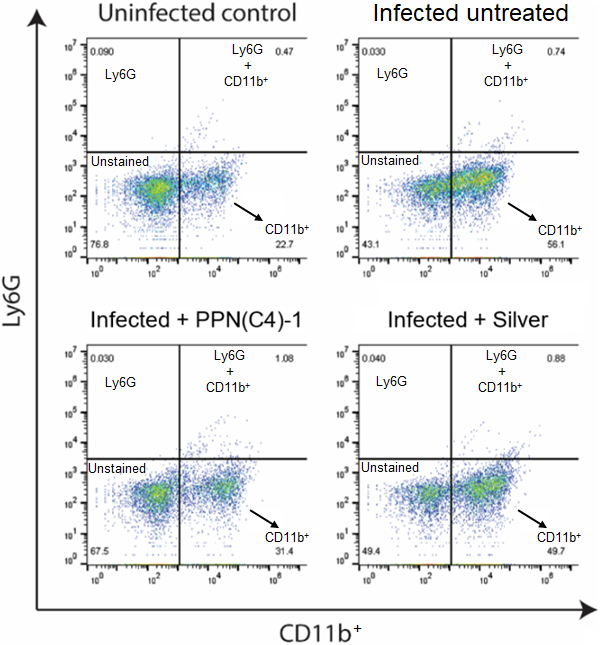
**

**Supplementary Figure 11.** FACS analysis showing the distributed populations of (x-axis) CD11b^+^ and (y-axis) Ly6G cells on the wounds of uninfected control, untreated MRSA-infected control, and MRSA-infected mice treated with PPN(C4)-1 and silver dressings on day 2 post-treatment.

**
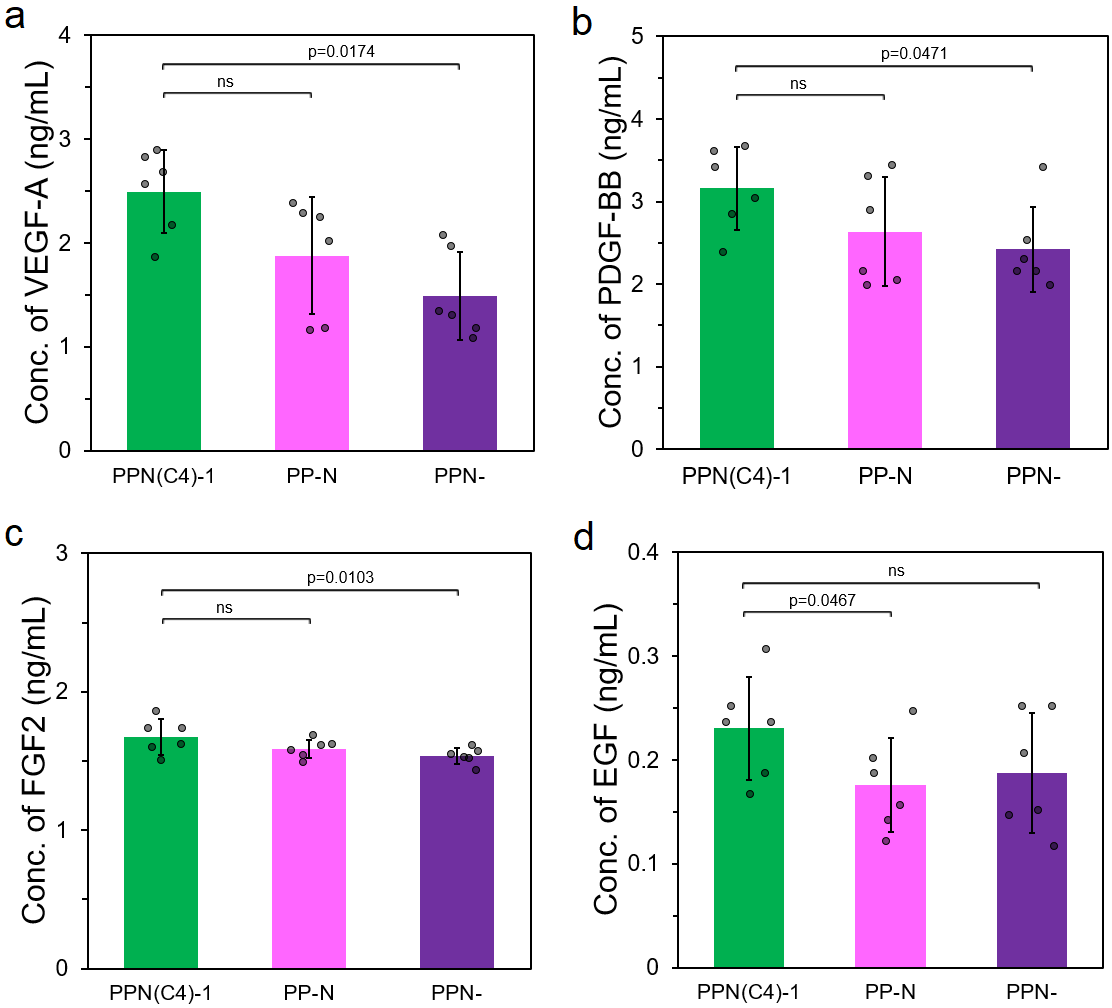
**

**Supplementary Figure 12.** Concentrations of wound healing factors (a) VEGF-A, (b) PDGF-BB, (c) FGF-2 and (d) EGF on MSRA USA300-infected wounds in diabetic mice (n=6 mice, two-tailed Student’s *t*-test, data are presented as mean values ± SD) after treatment for 2 days. PP-N is a PPN hydrogel without PIM(C4)-Mal, and PPN- is a PPN hydrogel without NAC.


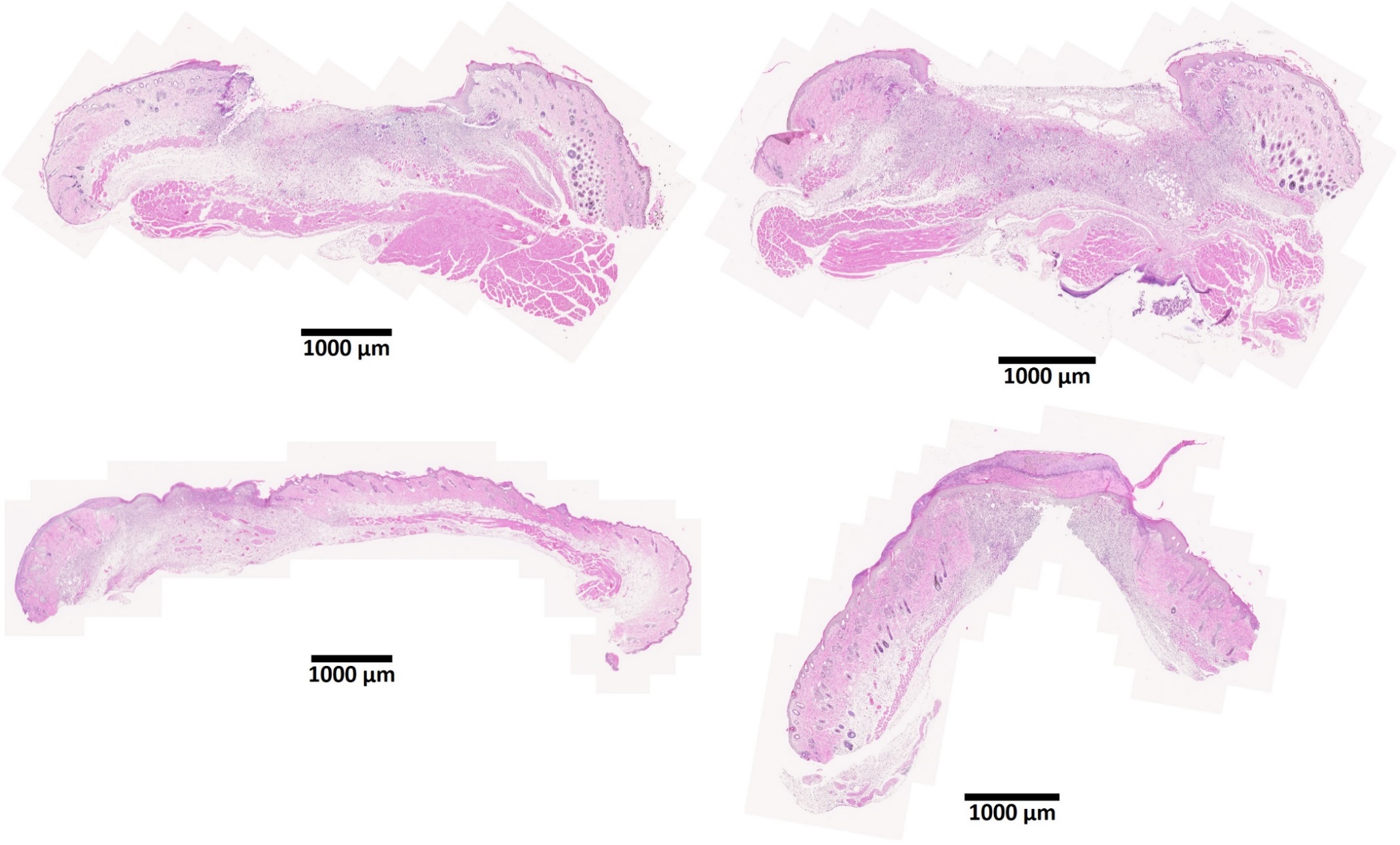


**Supplementary Figure 13a.** Histology images of H&E stained tissue sections of the untreated control wounds on day 7 post-treatment. Scale bar = 1 mm.


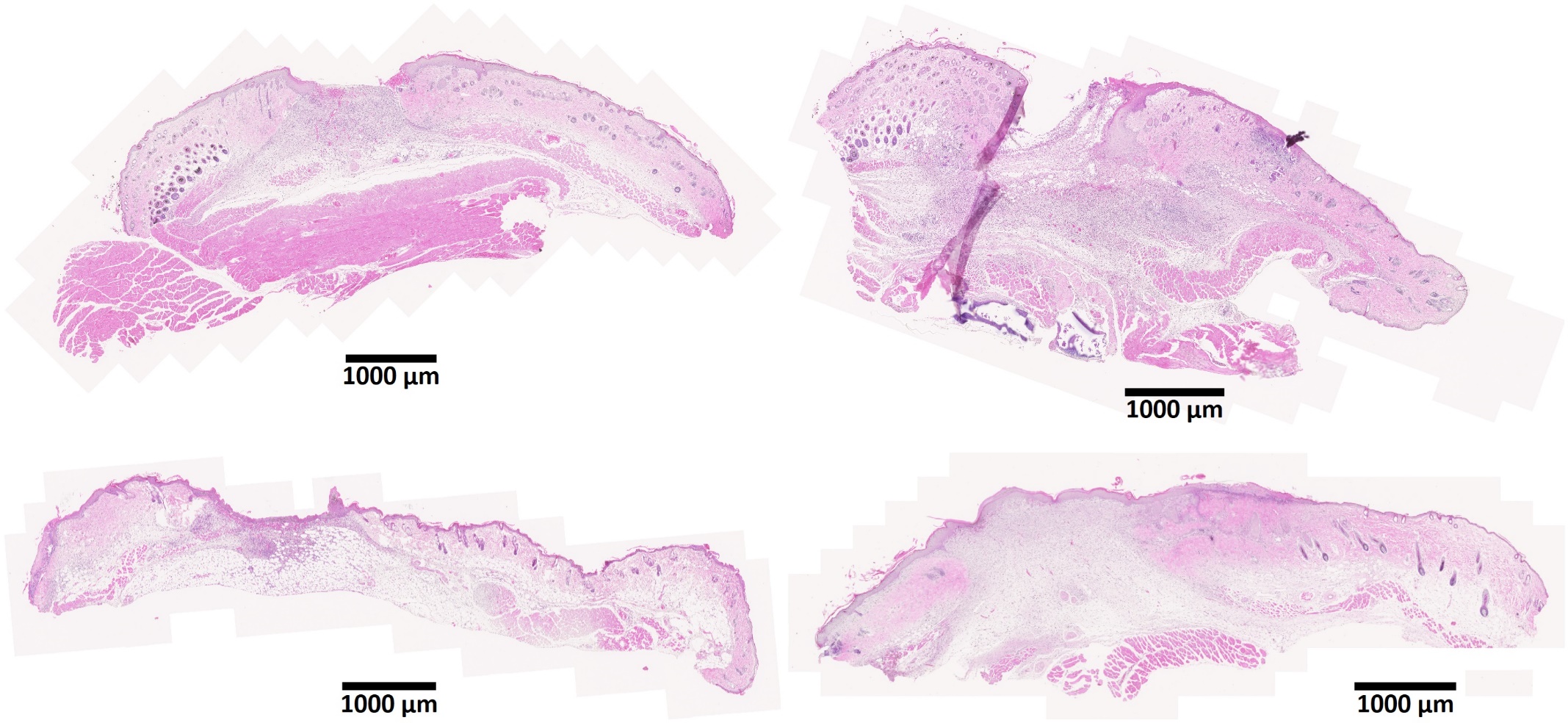


**Supplementary Figure 13b.** Histology images of H&E stained tissue sections of the PPcontrol hydrogel-treated wounds on day 7 post-treatment. Scale bar = 1 mm.


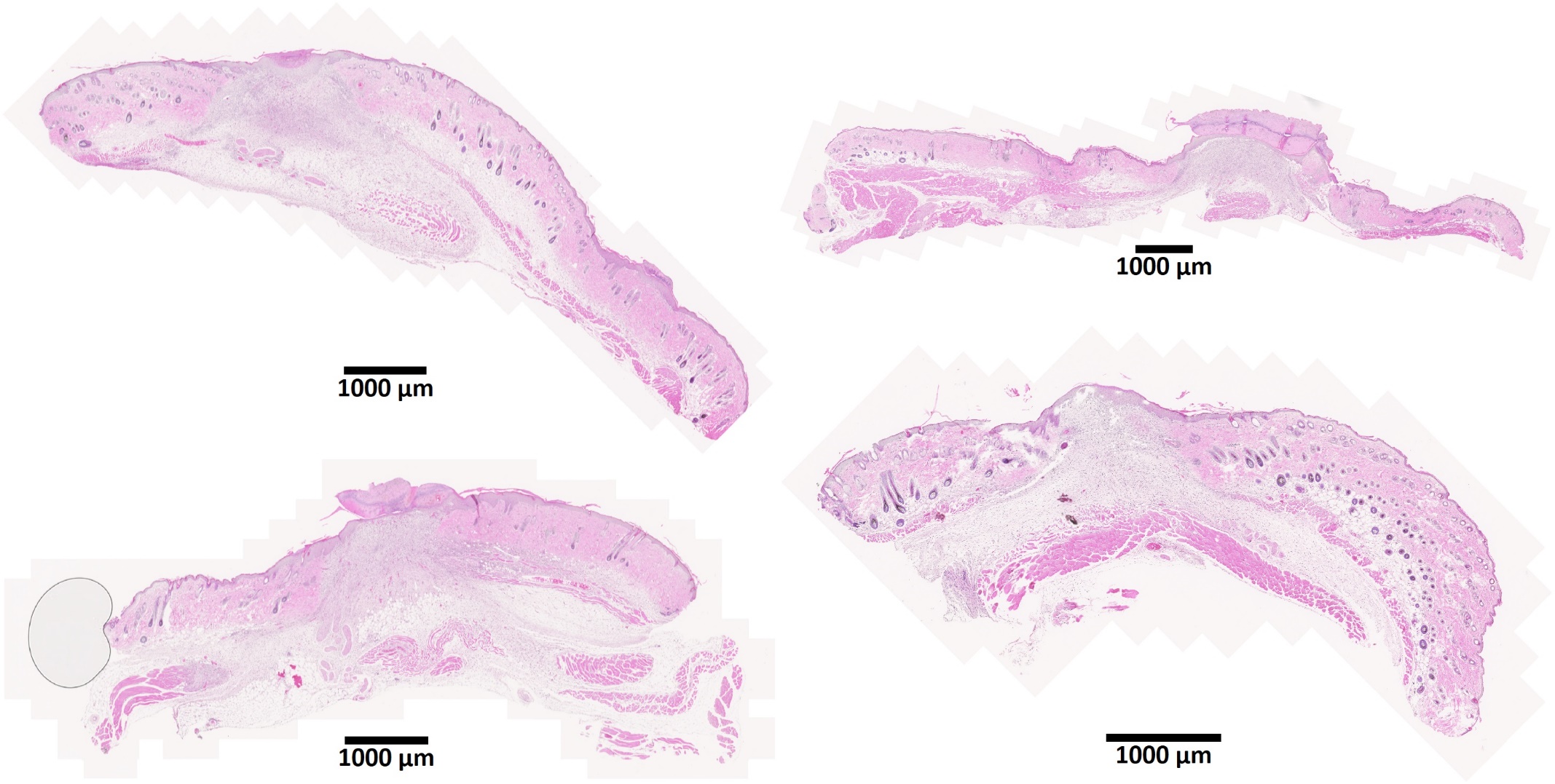


**Supplementary Figure 13c.** Histology images of H&E stained tissue sections of the silver hydrogel-treated wounds on day 7 post-treatment. Scale bar = 1 mm.


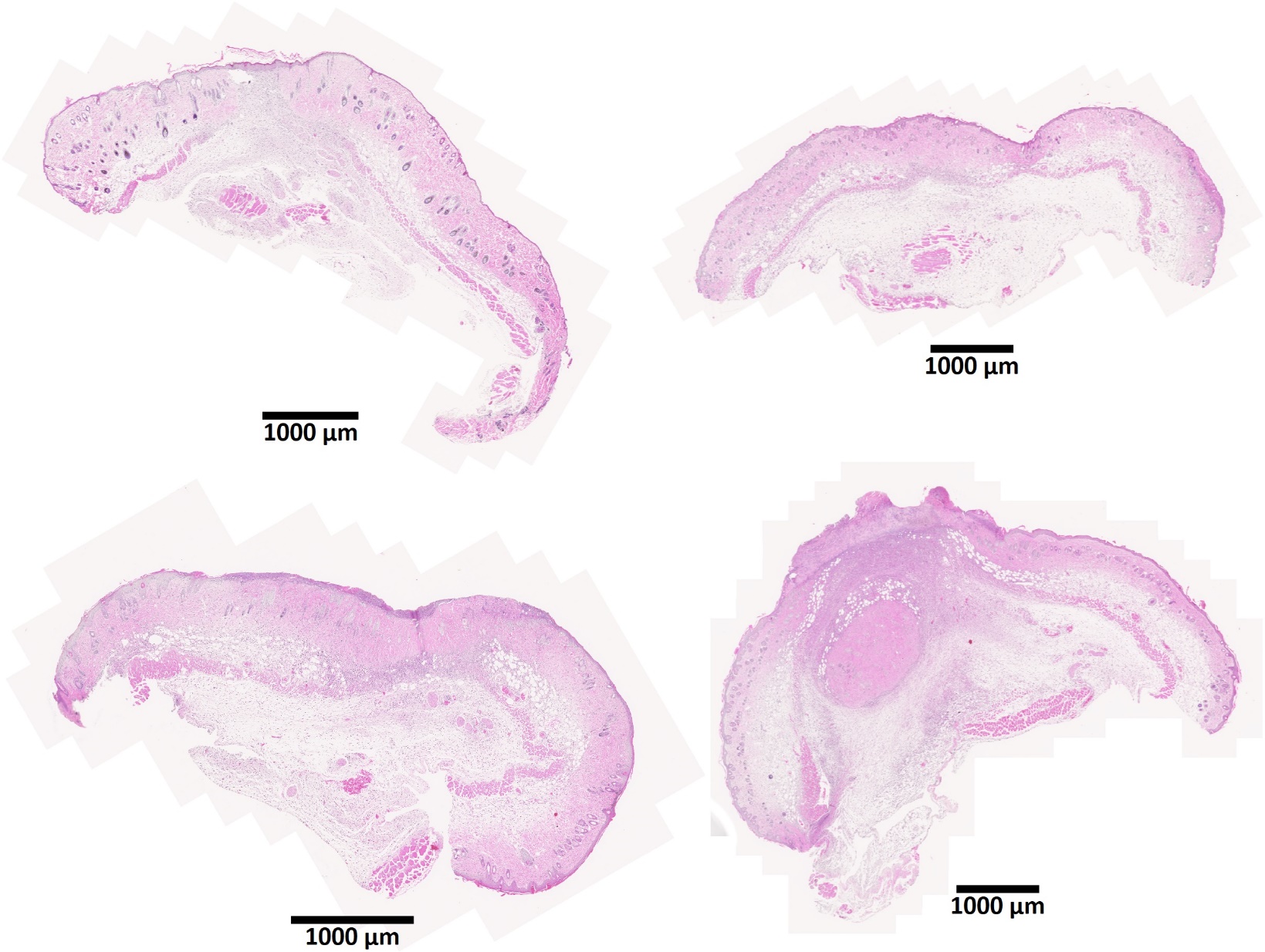


**Supplementary Figure 13d.** Histology images of H&E stained tissue sections of the PPN(C4)-1 hydrogel-treated wounds on day 7 post-treatment. Scale bar = 1 mm.


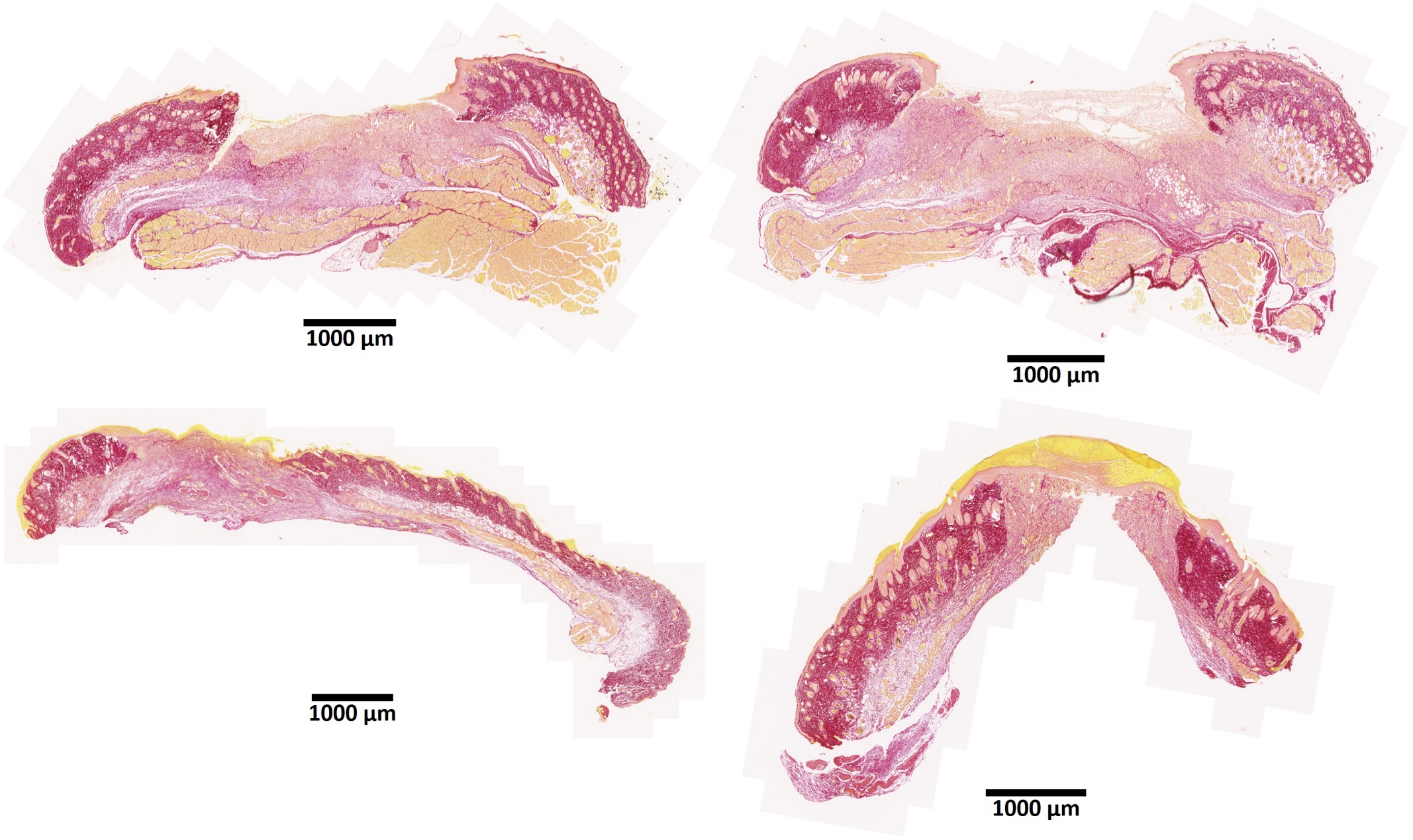


**Supplementary Figure 14a.** Histology images of picrosirius red stained tissue sections of the untreated control wounds on day 7 post-treatment. Scale bar = 1 mm.


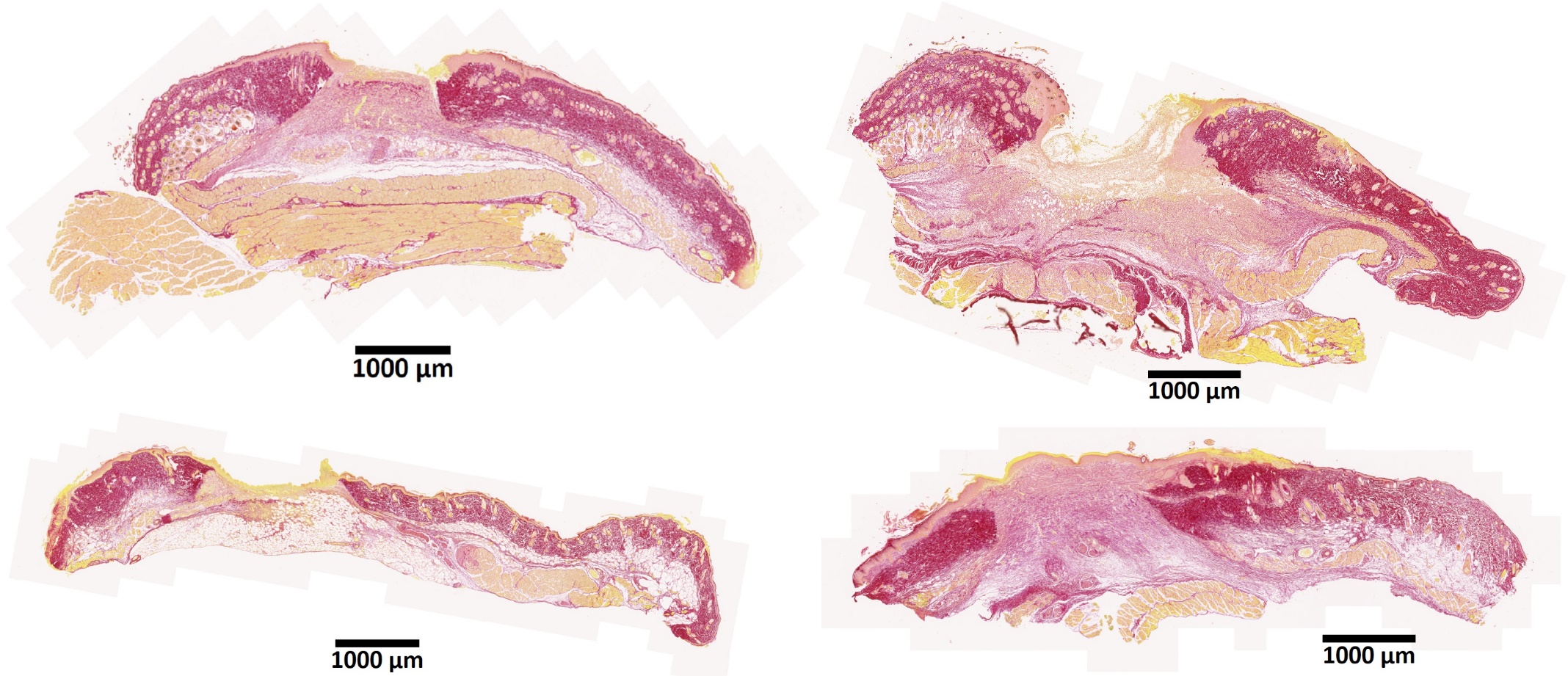


**Supplementary Figure 14b.** Histology images of picrosirius red stained tissue sections of the PPcontrol hydrogel-treated wounds on day 7 post-treatment. Scale bar = 1 mm.


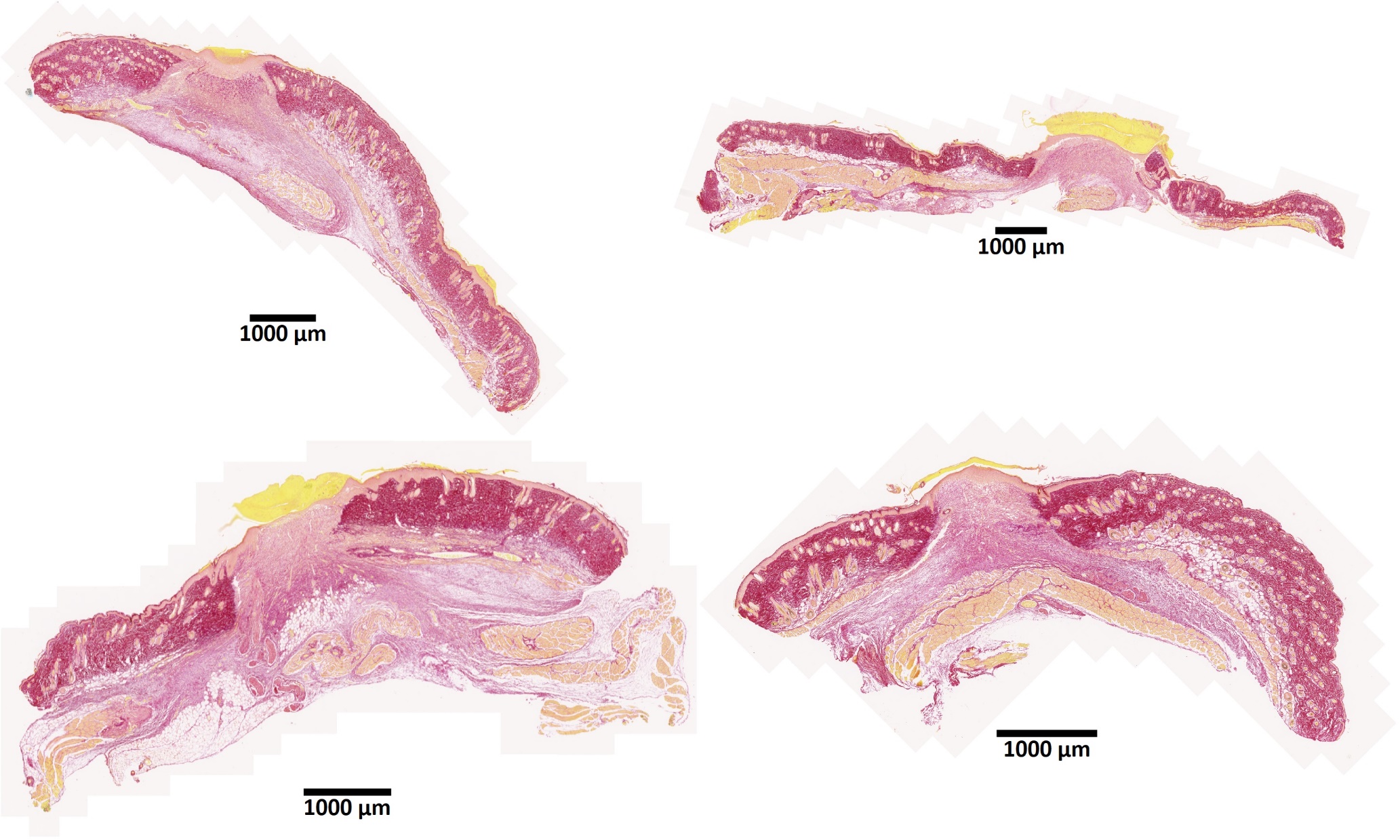


**Supplementary Figure 14c.** Histology images of picrosirius red stained tissue sections of the silver hydrogel-treated wounds on day 7 post-treatment. Scale bar = 1 mm.


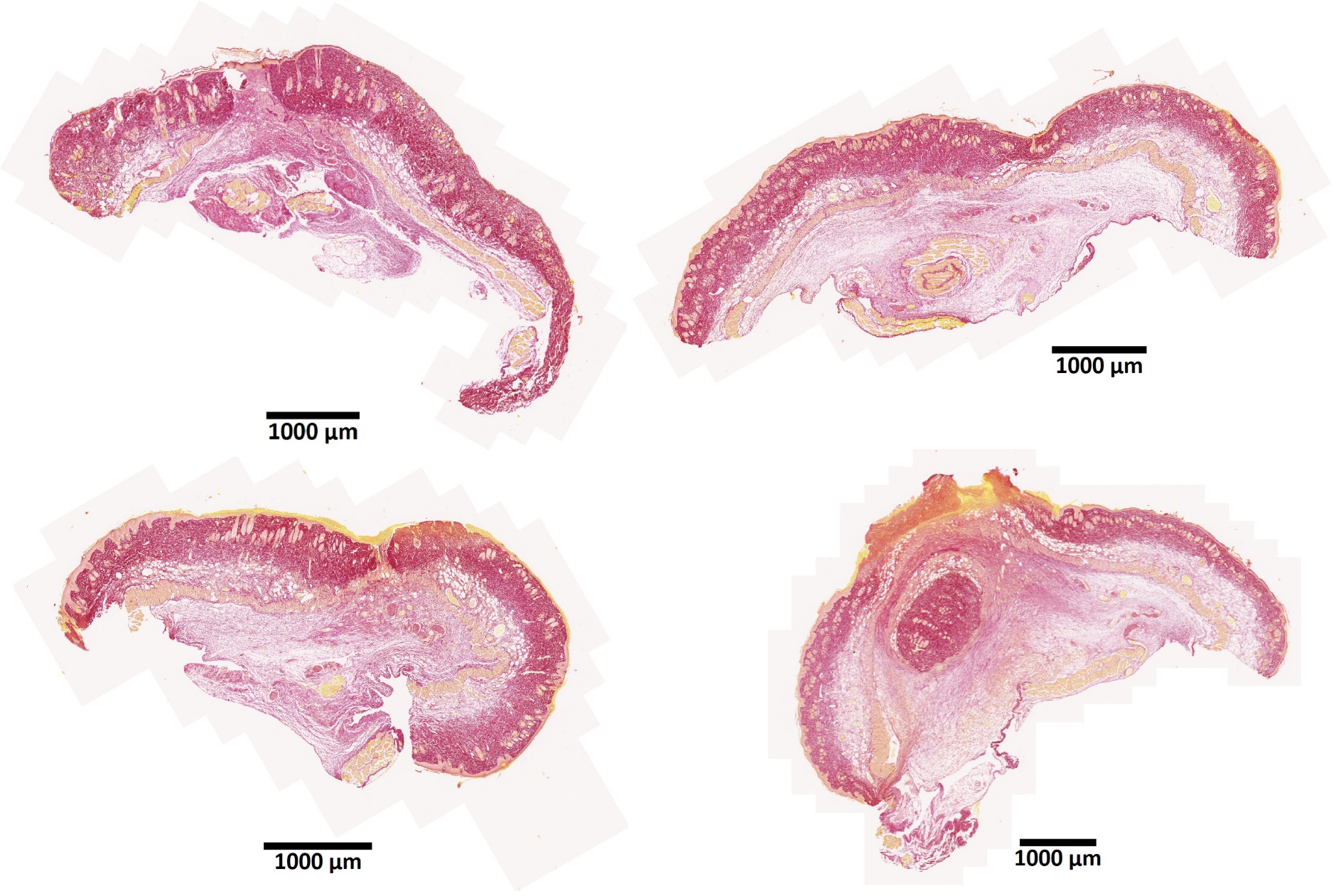


**Supplementary Figure 14d.** Histology images of picrosirius red stained tissue sections of the PPN(C4)-1 hydrogel-treated wounds on day 7 post-treatment. Scale bar = 1 mm.


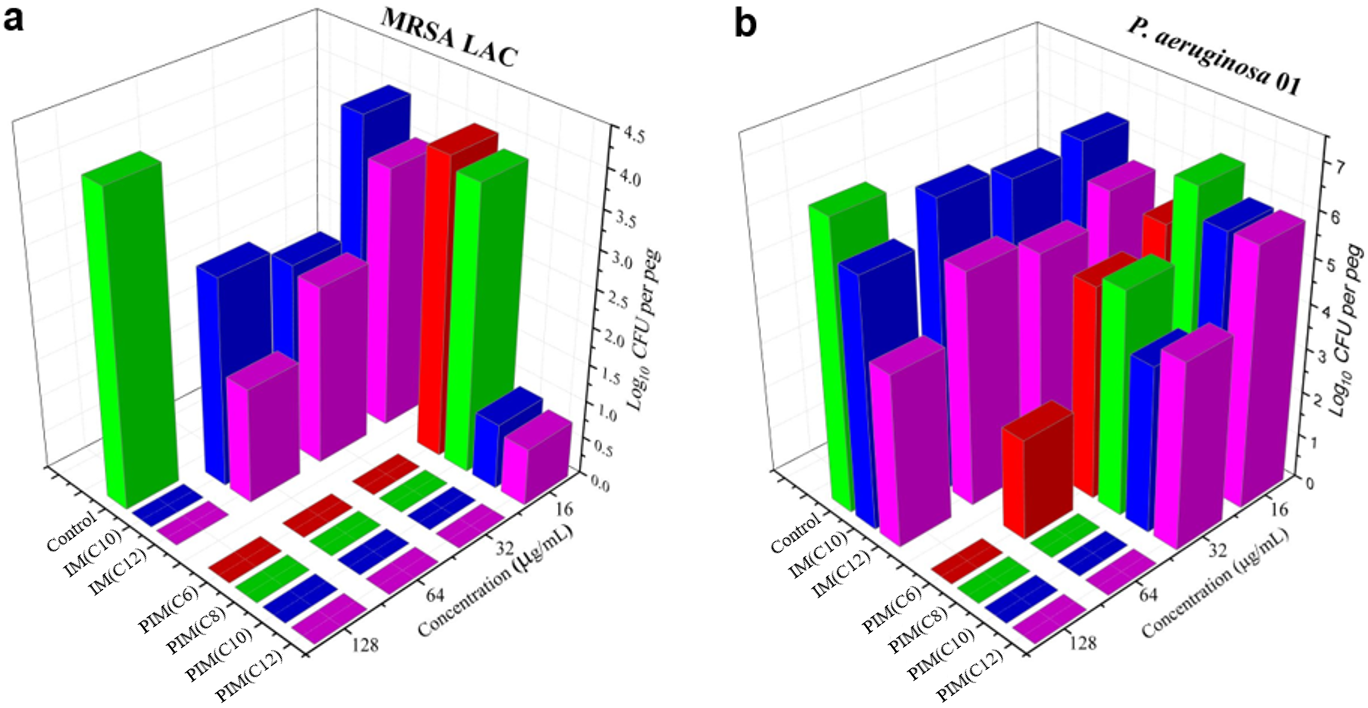


**Supplementary Figure 15.** Antibiofilm properties of imidazolium (IM(Cn)) and polyimidazolium (PIM(Cn)) compounds measured by minimum biofilm eradication concentration (MBEC) assay, showing viable bacterial counts of (a) MRSA LAC and (b) *P. aeruginosa* 01 (PAO1) on each microtiter plate peg after 4 h of treatment with IM(Cn) and PIM(Cn) compounds.


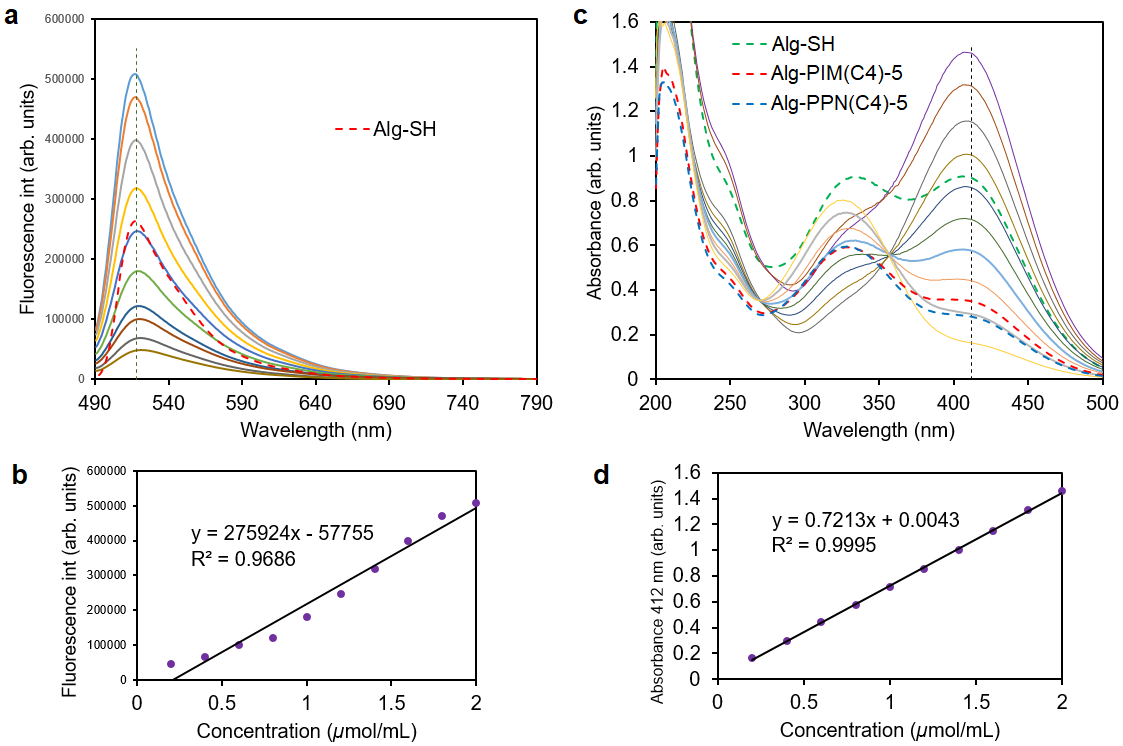


**Supplementary Figure 16.** Determination of the thiol contents of Alg-SH based on the reaction of the thiol functionalities with (a,b) F5M dye and (c,d) DTNB pro-dye. (a) Fluorescence spectra of the F5M-labelled Alg-SH (dashed line) as compared to the F5M-labelled cysteine standards (solid lines). (b) Intensity calibration of F5M-labelled cysteine standards. (c) Absorbance spectra of DTNB-reacted Alg-SH, Alg-PIM(C4)-5 and Alg-PPN(C4)-5 compared to DTNB-reacted cysteine standards (solid lines). (d) Intensity calibration standard of DTNB-reacted cysteine standards.

**
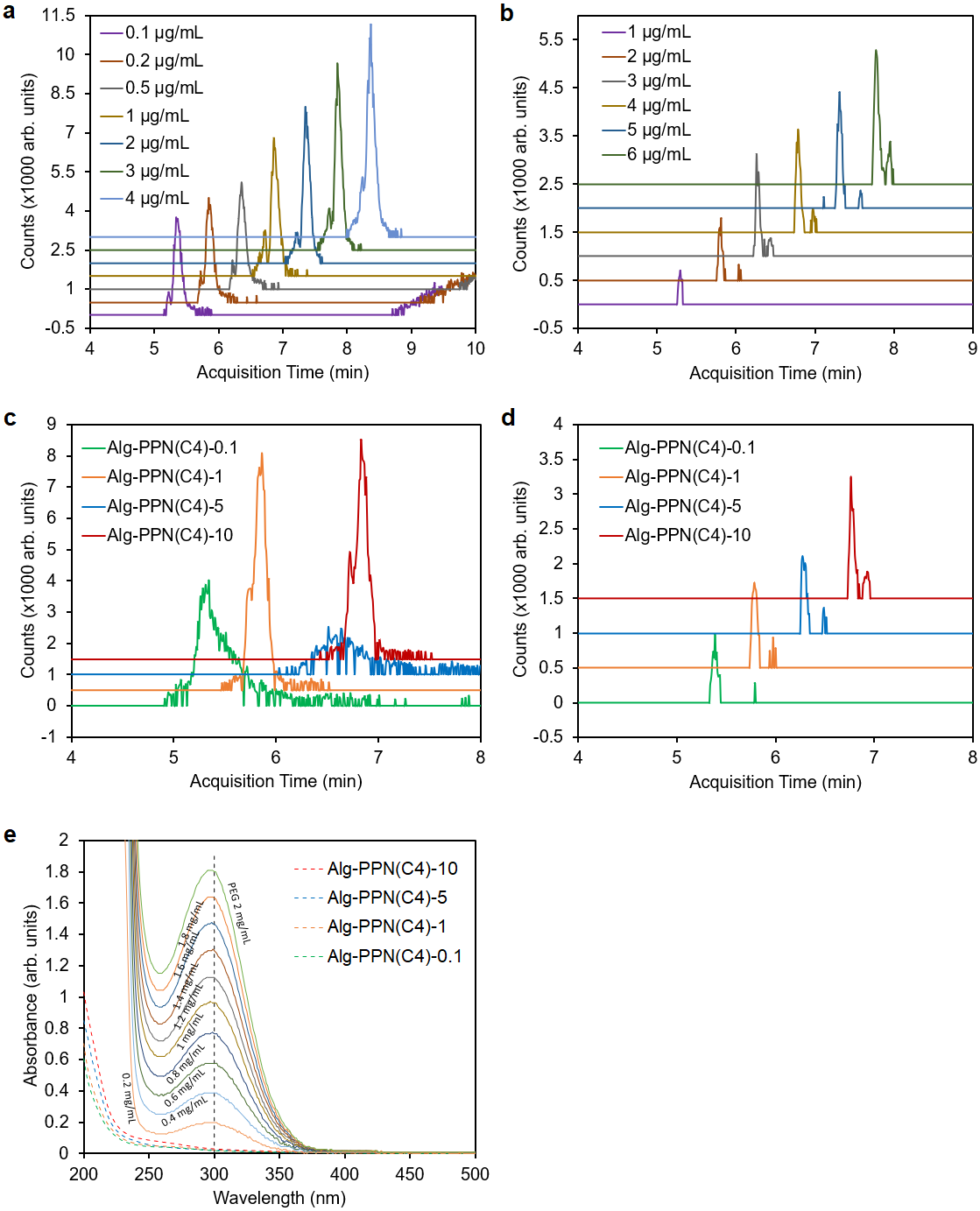
**

**Supplementary Figure 17.** (a-d) Extracted ion chromatograms of (a) PIM(C4) and (b) NAC calibration standards, and (c, d) supernatants after incubation with Alg-PIM(C4) fibers at 37 °C for 24 h, measured at *m*/*z* of (a, c) 245.1760 for PIM(C4) and (b, d) 164.0376 for NAC. (e) Absorbance spectra of the supernatants after incubation with Alg-PPN(C4) fibers at 37 °C for 24 h (dashed lines) compared with the calibration absorbance of PEG-4Mal component (solid lines). Calibration spectra (solid lines) are labelled with PEG-4Mal component concentrations.

**
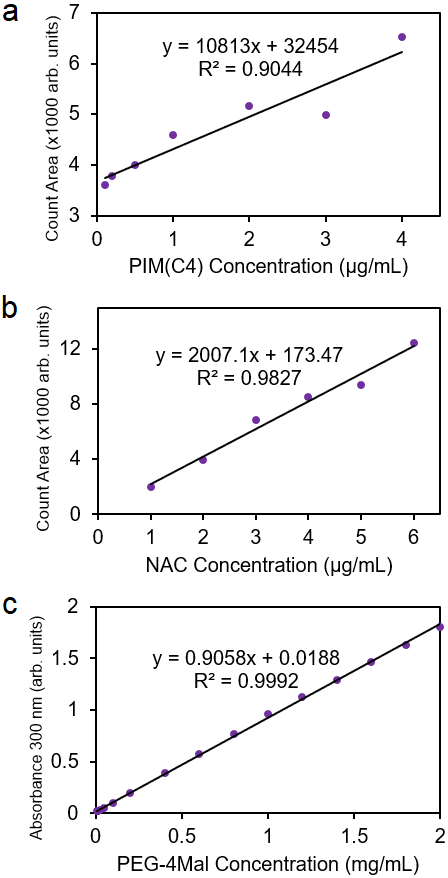
**

**Supplementary Figure 18.** Calibration curves of (a, b) count area and (c) absorbance versus concentration for (a) PIM(C4), (b) NAC and (c) PEG-4Mal standards. The calibration curves were obtained from the calibration spectra in Figures S15 (a, b, e).


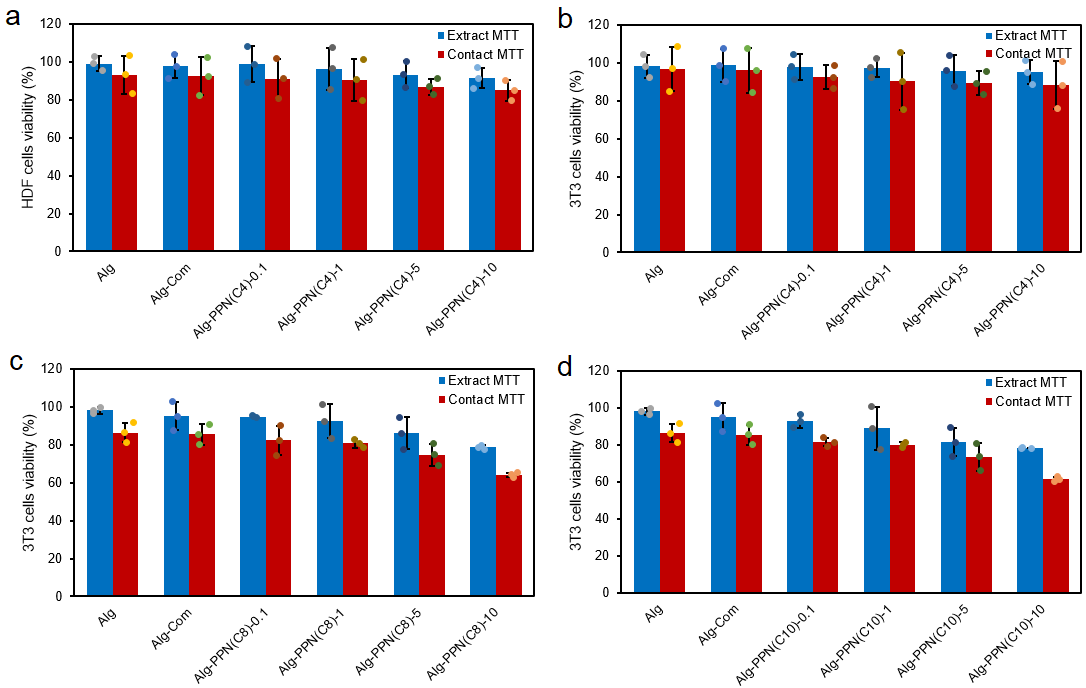


**Supplementary Figure 19.** Cell viability of (a) HDF and (b-d) mouse 3T3 fibroblasts after incubation with the extract supernatant (blue) or direct contact immersion (red) of (a,b) Alg-PPN-C4, (c) Alg-PPN-C8, and (d) Alg-PPN-C10 fibers at 37 °C for 24 h (n=3 cells examined over 3 independent experiments, data are presented as mean values ± SD).


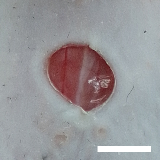

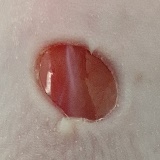

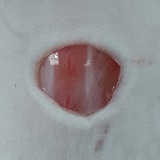

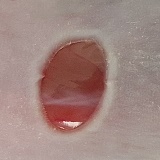

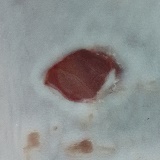

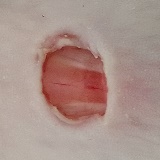

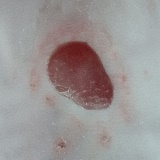

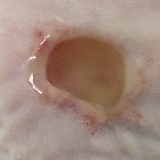

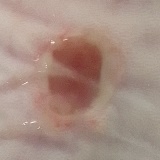

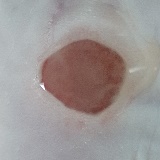

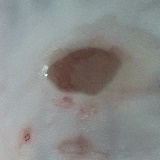

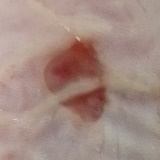

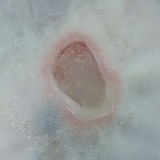

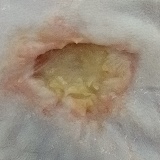

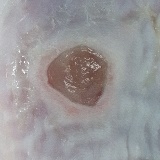

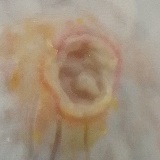

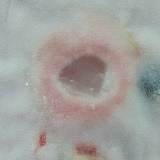

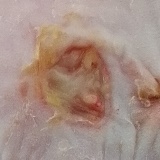

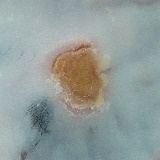

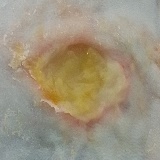

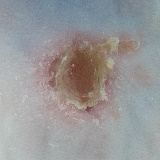

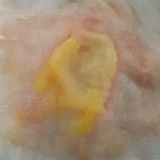

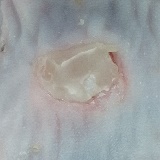

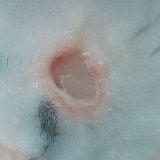

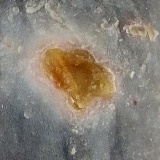

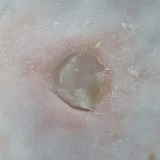

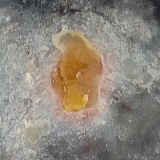

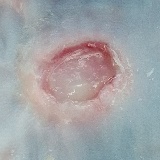

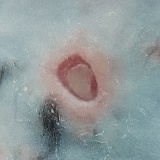

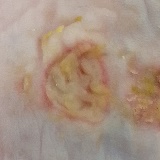

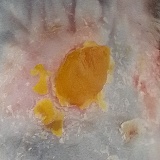

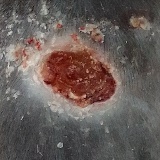

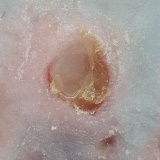

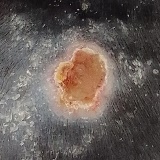

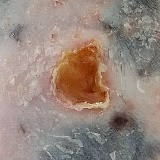

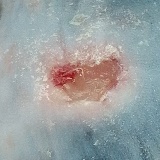

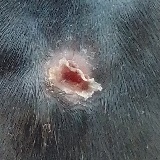

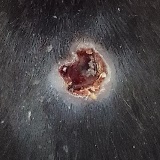

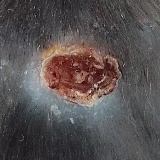

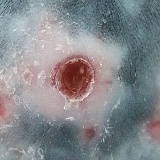

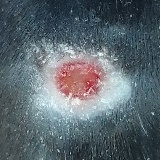

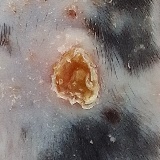

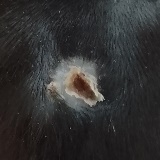

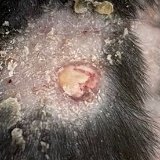

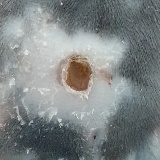

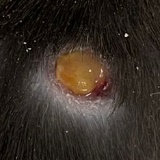

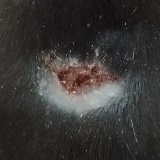

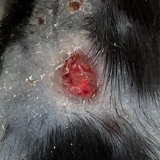


**Supplementary Figure 20a.** Visual appearance of untreated control wounds on CR-PA infected diabetic murine model between dressing changes over 2 weeks. Scale bar = 5 mm. (Six mice were used.)

Untreated

Untreated

Untreated

Untreated

Untreated

Untreated

a

Day 0

Day 1

Day 3

Day 5

Day 7

Day 9

Day 14

Day 12


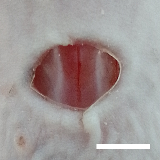

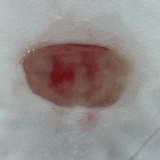

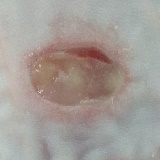

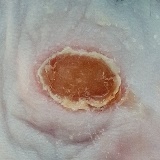

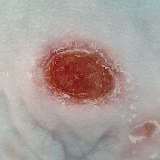

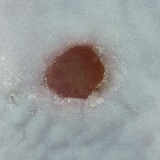

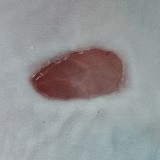

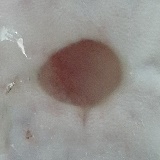

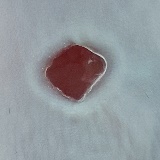

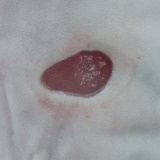

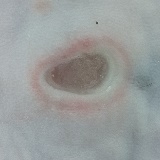


**Supplementary Figure 20b.** Visual appearance of Alg fibers-treated wounds on CR-PA infected diabetic murine model between dressing changes over 2 weeks. Scale bar = 5 mm. (Six mice and 1 batch of polymer were used.)

Alg

Alg

Alg

Alg

Alg

Alg

b

Day 0

Day 1

Day 3

Day 5

Day 7

Day 9

Day 14

Day 12

**Supplementary Figure 20c.** Visual appearance of Alg-Ag fibers-treated wounds on CR-PA infected diabetic murine model between dressing changes over 2 weeks. Scale bar = 5 mm. (Six mice and 1 batch of Alg-Ag fiber were used.)

Alg-Ag

Alg-Ag

Alg-Ag

Alg-Ag

Alg-Ag

Alg-Ag

c

Day 0

Day 1

Day 3

Day 5

Day 7

Day 9

Day 14

Day 12

**Supplementary Figure 20d.** Visual appearance of Alg-PPN fibers-treated wounds on CR-PA infected diabetic murine model between dressing changes over 2 weeks. Scale bar = 5 mm. (Six mice and 1 batch of polymers were used.)

Alg-PPN(C8)-5 / Alg-PPN(C4)-5

d

Day 0

Day 1

Day 3

Day 5

Day 7

Day 9

Day 14

Day 12

**Supplementary Figure 21.** (a) Percentage of Ly6G cells in CR-PA-infected wounds of diabetic mice (n=6 mice) after treatment for 2 days (two-tailed Student’s *t*-test, data are presented as mean values ± SD). Distributed populations of (x-axis) CD11b^+^ and (y-axis) Ly6G cells on the (b) untreated control, (c) Alg, (d) Alg-Ag, and (e) Alg-PPN fibers-treated wounds of the CR-PA-infected mice.

**Gating strategy for fluorescence activated cell sorting (FACS) flow cytometry**

Each sample had 4 different staining methods, *i.e.*: unstained, Ly6G only, CD11B^+^ only, and double stain (Ly6G and CD11B^+^). We based all our stained samples against their respective unstained samples, gating the unstained cells in the lower left quadrant. We used the previously determined quadrant for their stained counterparts. We started the analysis by using the forward and side scatter plots to group the live cells together under P1, avoiding the dead cells at the bottom left corner of the plot. These live cells were further distinguished into FL1 (Ly6G) and FL4 (CD11B^+^) to see where they fell into. All unstained cells were regarded as those in the lower left quadrant, all Ly6G positive cells were in the upper left quadrant, all CD11B^+^ positive cells were in the lower right quadrant, and all double positive cells were in the upper right quadrant.

**Supplementary Figure 22.** Exemplification of gating strategy for flow cytometry to acquire the distributed populations of (x-axis) CD11b^+^ and (y-axis) Ly6G cells on the uninfected control mouse.
