# Supplementary material for: Hydrogel dressings with intrinsic antibiofilm and antioxidative dual functionalities accelerate infected diabetic wound healing
Source: Nat Commun. 2024 Feb 1;15:954. doi: 10.1038/s41467-024-44968-y (PMC10830466; doi:10.1038/s41467-024-44968-y)
Supplement: Supplementary file 3 — Description of Additional Supplementary Files [file 41467_2024_44968_MOESM3_ESM.pdf]

Description of Additional Supplementary Files

File Name: Supplementary Movie 1
Description: Film Application on Mouse Wound.

File Name: Supplementary Movie 2
Description: Film Removal from Mouse Wound.

File Name: Supplementary Movie 3
Description: Fiber Application on Mouse Wound.

File Name: Supplementary Movie 4
Description: Fiber Removal from Mouse Wound.
