# Supplementary material for: Hydrogel dressings with intrinsic antibiofilm and antioxidative dual functionalities accelerate infected diabetic wound healing
Source: Nat Commun. 2024 Feb 1;15:954. doi: 10.1038/s41467-024-44968-y (PMC10830466; doi:10.1038/s41467-024-44968-y)
Supplement: Supplementary file 8 — Reporting Summary [file 41467_2024_44968_MOESM8_ESM.pdf]

nature portfolio

Corresponding author(s): Mary B. Chan-Park, Nguan Soon Tan

Last updated by author(s): Dec 24, 2023

Reporting Summary

Nature Portfolio wishes to improve the reproducibility of the work that we publish. This form provides structure for consistency and transparency in reporting. For further information on Nature Portfolio policies, see our Editorial Policies and the Editorial Policy Checklist.

Statistics

For all statistical analyses, confirm that the following items are present in the figure legend, table legend, main text, or Methods section.

n/a	Confirmed	
☐	☒	The exact sample size (n) for each experimental group/condition, given as a discrete number and unit of measurement
☐	☒	A statement on whether measurements were taken from distinct samples or whether the same sample was measured repeatedly
☐	☒	The statistical test(s) used AND whether they are one- or two-sided<br>*Only common tests should be described solely by name; describe more complex techniques in the Methods section.*
☒	☐	A description of all covariates tested
☐	☒	A description of any assumptions or corrections, such as tests of normality and adjustment for multiple comparisons
☐	☒	A full description of the statistical parameters including central tendency (e.g. means) or other basic estimates (e.g. regression coefficient) AND variation (e.g. standard deviation) or associated estimates of uncertainty (e.g. confidence intervals)
☐	☒	For null hypothesis testing, the test statistic (e.g. F, t, r) with confidence intervals, effect sizes, degrees of freedom and P value noted<br>*Give P values as exact values whenever suitable.*
☒	☐	For Bayesian analysis, information on the choice of priors and Markov chain Monte Carlo settings
☒	☐	For hierarchical and complex designs, identification of the appropriate level for tests and full reporting of outcomes
☒	☐	Estimates of effect sizes (e.g. Cohen's d, Pearson's r), indicating how they were calculated

Our web collection on statistics for biologists contains articles on many of the points above.

Software and code

Policy information about availability of computer code

Data collection	Tecan i-control (version 3.9.1.0), UV-Vis spectrophotometer (Shimadzu UV-1800), liquid chromatography-mass spectrometer (Agilent 6550 iFunnel Q-TOF), BD Accuri C6 Cytometer, LSM 800 confocal microscope (ZEISS), Axio Scan.Z1 microscope (ZEISS), Nikon SMZ745T microscope
Data analysis	Microsoft Excel (version 2016), ImageJ (version 2.1.0), FlowJo (version 7.6.5)

For manuscripts utilizing custom algorithms or software that are central to the research but not yet described in published literature, software must be made available to editors and reviewers. We strongly encourage code deposition in a community repository (e.g. GitHub). See the Nature Portfolio guidelines for submitting code & software for further information.

Data

Policy information about availability of data

All manuscripts must include a data availability statement. This statement should provide the following information, where applicable:

- Accession codes, unique identifiers, or web links for publicly available datasets
- A description of any restrictions on data availability
- For clinical datasets or third party data, please ensure that the statement adheres to our policy

The experimental data generated in this study are provided within the Article and Supplementary Information. All data are available from the corresponding authors upon request.

Research involving human participants, their data, or biological material

Policy information about studies with human participants or human data. See also policy information about sex, gender (identity/presentation), and sexual orientation and race, ethnicity and racism.

Reporting on sex and gender	N/A
Reporting on race, ethnicity, or other socially relevant groupings	N/A
Population characteristics	N/A
Recruitment	N/A
Ethics oversight	N/A

Note that full information on the approval of the study protocol must also be provided in the manuscript.

Field-specific reporting

Please select the one below that is the best fit for your research. If you are not sure, read the appropriate sections before making your selection.

☒ Life sciences ☐ Behavioural & social sciences ☐ Ecological, evolutionary & environmental sciences

For a reference copy of the document with all sections, see nature.com/documents/nr-reporting-summary-flat.pdf

Life sciences study design

All studies must disclose on these points even when the disclosure is negative.

Sample size	For the in vivo mouse experiments, an appropriate sample size (n = 6 mice per data point per treatment group, with 1 wound per mice) was used to provide statistical replicates. In a pilot experiment, n = 3 mice per data point (with fewer data points) was used for preliminary assessment of wound treatment.<br>For the ex vivo human skin equivalent experiment, an appropriate sample size (n ≥ 9 wounds per treatment group) was used to provide statistical replicates. These sample sizes were sufficient to investigate the efficacy of the treatment as compared to control group with significant difference.
Data exclusions	No data was excluded.
Replication	The in vivo mouse experiments were reliably reproduced by the pilot study and the actual experiment on the basis of bacterial count, wound size and closure. The tests were replicated twice and performed independently.
Randomization	All tests were performed with randomly allocated experimental groups, and samples were analyzed together in each experiment.
Blinding	Data acquisition and analysis were performed by investigators blinded to the groups.

Reporting for specific materials, systems and methods

We require information from authors about some types of materials, experimental systems and methods used in many studies. Here, indicate whether each material, system or method listed is relevant to your study. If you are not sure if a list item applies to your research, read the appropriate section before selecting a response.

Materials & experimental systems

n/a	Involved in the study	
☐	☒	Antibodies
☐	☒	Eukaryotic cell lines
☒	☐	Palaeontology and archaeology
☐	☒	Animals and other organisms
☒	☐	Clinical data
☒	☐	Dual use research of concern
☒	☐	Plants

Methods

n/a	Involved in the study	
☒	☐	ChIP-seq
☐	☒	Flow cytometry
☒	☐	MRI-based neuroimaging

Antibodies

Antibodies used	Mouse pro-MMP9 (EMMMP9), VEGF-A (BMS619-2), PDGF-BB (BMS2071), FGF-2 (EMFGF2), and EGF (EMEGF) were purchased from

	Thermo Fisher Sci. Inc. (Waltham, MA). CD11b+ (130-113-231) and Ly6G (130-102-296) antibodies were purchased from Miltenyi Biotec. Mouse monoclonal anti-K10 (DE-K10) was purchased from Dako (DKO.M7002). Mouse monoclonal anti-K14 (LL001) was a supernatant from E. Birgitte Lane's lab (A*STAR). Mouse monoclonal anti-p63 (4A4) was purchased from Abcam (Ab735).
Validation	All antibodies are commercially available and have been tested or validated by the manufacturers (as in their webpages below).<br>Pro-MMP9: https://www.thermofisher.com/elisa/product/pro-MMP-9-Mouse-ELISA-Kit/EMMMP9<br>VEGF-A: https://www.thermofisher.com/elisa/product/VEGF-A-Mouse-ELISA-Kit/BMS619-2<br>PDGF-BB: https://www.thermofisher.com/elisa/product/Mouse-PDGF-BB-ELISA-Kit/EM63RB<br>FGF-2: https://www.thermofisher.com/elisa/product/FGF2-bFGF-Mouse-ELISA-Kit/EMFGF2<br>EGF: https://www.thermofisher.com/elisa/product/EGF-Mouse-ELISA-Kit/EMEGF<br>CD11b+: https://www.miltenyibiotec.com/SG-en/products/cd11b-antibody-anti-human-mouse-m1-70-15-11-5.html#apc<br>Ly6G: https://www.miltenyibiotec.com/SG-en/products/ly-6g-antibody-anti-mouse-1a8.html#fitc<br>anti-K10: https://www.labome.com/product/Dako/M7002.html<br>anti-K14: https://www.labome.com/product/Santa-Cruz-Biotechnology/sc-53253.html<br>anti-p63: https://www.abcam.com/p63-antibody-4a4-ab735.html

Eukaryotic cell lines

Policy information about cell lines and Sex and Gender in Research

Cell line source(s)	NIH/3T3 (ATCC CRL-1658) mouse fibroblast cells were purchased from American Type Culture Collection (ATCC, Manassas, VA). Human dermal fibroblast cells (NHDF-Ad, CC-2511) were purchased from Lonza (Basel, Switzerland).
Authentication	All cell lines are commercially available and have been tested or authenticated by the manufacturers on the basis of standard techniques including morphology check, isoenzyme analysis and mycoplasma detection (as in their webpages below).<br>3T3: https://www.atcc.org/products/crl-1658<br>NHDF-Ad: https://bioscience.lonza.com/lonza_bs/SG/en/Primary-and-Stem-Cells/p/000000000000184914/NHDF-Ad---Human-Dermal-Fibroblasts%2C-Adult
Mycoplasma contamination	Mycoplasma contamination was not detected.
Commonly misidentified lines<br>(See ICLAC register)	No commonly misidentified cell line was used in this study.

Animals and other research organisms

Policy information about studies involving animals; ARRIVE guidelines recommended for reporting animal research, and Sex and Gender in Research

Laboratory animals	Eight-week old male C57BL/6J mice were purchased from InVivos Pte. Ltd. (Singapore). Mice were housed in a 12-h light/12-h dark cycle under ambient temperature and humidity, with access to food and water.
Wild animals	No wild animal was involved in this study.
Reporting on sex	No report was conclusive that the findings apply to only one sex.
Field-collected samples	No field-collected sample was involved in this study.
Ethics oversight	All mouse studies were approved and performed in compliance with the regulations of the Institutional Animal Care and Use Committee (IACUC) of Nanyang Technological University (NTU) under protocols numbers A18051 and A21023.

Note that full information on the approval of the study protocol must also be provided in the manuscript.

Plants

Seed stocks	N/A
Novel plant genotypes	N/A
Authentication	N/A

Flow Cytometry

Plots

Confirm that:

- [x] The axis labels state the marker and fluorochrome used (e.g. CD4-FITC).
- [x] The axis scales are clearly visible. Include numbers along axes only for bottom left plot of group (a 'group' is an analysis of identical markers).
- [x] All plots are contour plots with outliers or pseudocolor plots.
- [x] A numerical value for number of cells or percentage (with statistics) is provided.

Methodology

Sample preparation	Diabetic C57BL/6J mouse skin lesion with bacteria was excised from the mouse. Lesion samples were dissociated using a Gentle MACS Dissociator (Miltenyi Biotec), filtered with 70 µm filter unit into single cells, and stained with CD11b+ and Ly6G antibodies for 30 minutes. Cells were then washed and reconstituted in PBS to start analysis in Flow Cytometer.
Instrument	BD Accuri C6 Flow Cytometer (BD Biosciences)
Software	FlowJo software version 7.6.5 (Tree Star)
Cell population abundance	Each sample contained more than 1 million cells in 200 µL suspension. At least 200,000 cells per sample were analyzed in BD Accuri Cytometer. Cells population were of skin origin as well as the hydrogel and bacteria used in the experimental set up. For unstained samples, majority of cells were found in the lower left quadrant. For Ly6G stained samples, an equal amount were found in the upper and lower left quadrants. For CD11B+ stained samples, majority of cells were found in the lower right quadrant. For double stained samples, the cells distributions were split among the lower left, lower right and upper right quadrants.
Gating strategy	Each sample had 4 different staining methods, i.e.: unstained, Ly6G only, CD11B+ only, and double stain (Ly6G and CD11B+). We based all our stained samples against their respective unstained samples, gating the unstained cells in the lower left quadrant. We used the previously determined quadrant for their stained counterparts. We started the analysis by using the forward and side scatter plots to group the live cells together under P1, avoiding the dead cells at the bottom left corner of the plot. These live cells were further distinguished into FL1 (Ly6G) and FL4 (CD11B+) to see where they fell into. All unstained cells were regarded as those in the lower left quadrant, all Ly6G positive cells were in the upper left quadrant, all CD11B+ positive cells were in the lower right quadrant, and all double positive cells were in the upper right quadrant.

- [x] Tick this box to confirm that a figure exemplifying the gating strategy is provided in the Supplementary Information.
